# Osteoporosis in Relation to a Bone-Related Aging Biomarker Derived from the Urinary Proteomic Profile: A Population Study

**DOI:** 10.14336/AD.2024.0303

**Published:** 2024-03-03

**Authors:** Yu-Ling Yu, Dries S. Martens, De-Wei An, Babangida Chori, Agnieszka Latosinska, Justyna Siwy, Augustine N. Odili, Katarzyna Stolarz-Skrzypek, Gladys E. Maestre, Kei Asayama, Yan Li, Peter Verhamme, Karel Allegaert, Harald Mischak, Tim S. Nawrot, Jan A. Staessen

**Affiliations:** ^1^Research Unit Environment and Health, KU Leuven Department of Public Health and Primary Care, University of Leuven, Leuven, Belgium.; ^2^Non-Profit Research Association Alliance for the Promotion of Preventive Medicine, Mechelen, Belgium.; ^3^Centre for Environmental Sciences, Hasselt University, Hasselt, Belgium.; ^4^Department of Cardiovascular Medicine, Shanghai Key Laboratory of Hypertension, Shanghai Institute of Hypertension, State Key Laboratory of Medical Genomics, National Research Center for Translational Medicine, Ruijin Hospital, Shanghai Jiaotong University School of Medicine, Shanghai, China.; ^5^Doctoral School for Health and Life Sciences, Hasselt University, Diepenbeek, Belgium.; ^6^Center for Environmental Sciences, Hasselt University, Diepenbeek, Belgium.; ^7^Department of Medicine, Federal Medical Center Jabi, Abuja, Nigeria.; ^8^Mosaiques Diagnostics GmbH, Hannover, Germany.; ^9^First Department of Cardiology, Interventional Electrocardiology and Hypertension, Jagiellonian University, Kraków, Poland.; ^10^Department of Neurosciences and Department of Human Genetics, University of Texas Rio Grande Valley School of Medicine, Brownsville, Texas, USA.; ^11^Alzheimer’s Disease Resource Center for Minority Aging Research, University of Texas Rio Grande Valley, Brownsville, Texas, US.; ^12^Department of Hygiene and Public Health, Teikyo University School of Medicine, Tokyo, Japan.; ^13^Research Unit Hypertension and Cardiovascular Epidemiology, KU Leuven Department of Cardiovascular Sciences, University of Leuven, Leuven, Belgium.; ^14^Center for Molecular and Vascular Biology, KU Leuven Department of Cardiovascular Sciences, University of Leuven, Leuven, Belgium.; ^15^Department of Pharmaceutical and Pharmacological Sciences, KU Leuven, Leuven, Belgium.; ^16^KU Leuven Department of Development and Regeneration, KU Leuven, Leuven, Belgium.; ^17^Department of Hospital Pharmacy, Erasmus Medical Center, Rotterdam, the Netherlands.; ^18^Biomedical Sciences Group, Faculty of Medicine, University of Leuven, Leuven, Belgium.

**Keywords:** Aging, biomarker, bone, osteoporosis, urinary proteomics

## Abstract

Screening for and prevention of osteoporosis and osteoporotic fractures is imperative, given the high burden on individuals and society. This study constructed and validated an aging-related biomarker derived from the urinary proteomic profile (UPP) indicative of osteoporosis (UPPost-age). In a prospective population study done in northern Belgium (1985-2019), participants were invited for a follow-up examination in 2005-2010 and participants in the 2005-2010 examination again invited in 2009-2013. Participants in both the 2005-2010 and 2009-2013 examinations (*n* = 519) constituted the derivation (2005-2016 data) and time-shifted validation (2009-2013 data) datasets; 187 participants with only 2005-2010 data formed the synchronous validation dataset. The UPP was assessed by capillary electrophoresis coupled with mass spectrometry. Analyses focused on 2372 sequenced urinary peptides (101 proteins) with key roles in maintaining the integrity of bone tissue. In multivariable analyses with correction for multiple testing, chronological age was associated with 99 urinary peptides (16 proteins). Peptides derived from IGF2 and MGP were upregulated in women compared to men, whereas COL1A2, COL3A1, COL5A2, COL10A1 and COL18A1 were downregulated. Via application of a 1000-fold bootstrapped elastic regression procedure, finally, 29 peptides (10 proteins) constituted the UPPost-age biomarker, replicated across datasets. In cross-sectional analyses of 2009-2013 data (*n* = 706), the body-height-to-arm-span ratio, an osteoporosis marker, was negatively associated with UPPost-age (*p*<0.0001). Over 4.89 years (median), the 10-year risk of osteoporosis associated with chronological age and UPPost-age (53 cases including 37 fractures in 706 individuals) increased by 21% and 36% (*p* ≤ 0.044). Among 357 women, the corresponding estimates were 55% and 60% for incident osteoporosis (37 cases; *p* ≤ 0.0003) and 42% and 44% for osteoporotic fractures (25 cases; *p* ≤ 0.017). In conclusion, an aging-related UPP signature with focus on peptide fragments derived from bone-related proteins is associated with osteoporosis risk and available for clinical and trial research.

## INTRODUCTION

Osteoporosis is an age-related skeletal disorder characterized by the loss of bone mass, induced by increased osteoclast-stimulated bone resorption and inhibition of osteoblast-stimulated bone formation, and leading to the fragility of bone tissue and high risk of fractures [1]. Based on National Health and Nutrition Examination Survey 2005-2010, the prevalence of osteoporosis in American adults aged 50 years or older was 10.3%, translating into 10.2 million people at risk [2]. With increasing age the prevalence of osteoporosis raised exponentially in Americans, from 5.1% at age 50-59 years to 26.2% in octogenarians and the very elderly [2]. Moreover, 71% of osteoporotic fracture occurred among women [3], who had a higher prevalence of osteoporosis and osteoporotic fracture risk at any given age compared with men [2,4]. Because osteoporosis and its related fractures worsen quality of life [5] and represent a high socio-economic burden [3,6,7], early screening and prevention are imperative [8].

Urine contains more than 5000 sequenced peptides, which identify the parental proteins and in the case of osteoporosis reveal the dysregulated molecular features in the skeletal system [9,10]. Moreover, biological age, evaluated by standardized physiological measurements, functional tests or sophisticated “omics” technologies [11], allows a better prediction of the functional capacity later in life than chronological age. Previous studies demonstrated that the urinary peptidomic profile (UPP) is an effective predictor aging-associated disabilities [9]. However, few studies addressed how osteoporosis is related to the aging-related UPP signature [12]. Thus, the aims of the current study were to construct an aging-related biomarker indicative of osteoporosis (UPPost-age) and to validate the UPPost-age in the family-based Flemish Study on Environment, Genes, and Health Outcomes (FLEMENGHO) [9].

## MATERIAL AND METHODS

### Study population

FLEMENGHO complies with the Helsinki declaration and is registered with the Belgian Data Protection Authority (III 11/1234/13; Aug 22, 2013). The ethics committee of the University Hospitals Leuven, Belgium, approved the secondary use of FLEMENGHO data (B32220083510). From August 20, 1985, to December 14, 1990, a random sample of the households living in a geographically defined area of northern Belgium was investigated with the goal to recruit an equal number of participants in each of six subgroups stratified by sex and age (20-39 years, 40-59 years, and ≥60 years). All household members aged 20 years or older were invited to take part, provided that the quota of their sex-age group had not yet been met. From April 3, 1996, to May 12, 2007, recruitment of families continued, including young people aged 10-19 years. Participants younger than 18 years provided informed assent and their parents or custodians gave informed consent. Of 4286 people invited to participate in FLEMENGHO, 3343 consented (participation rate 78.0%).

**Figure 1.**
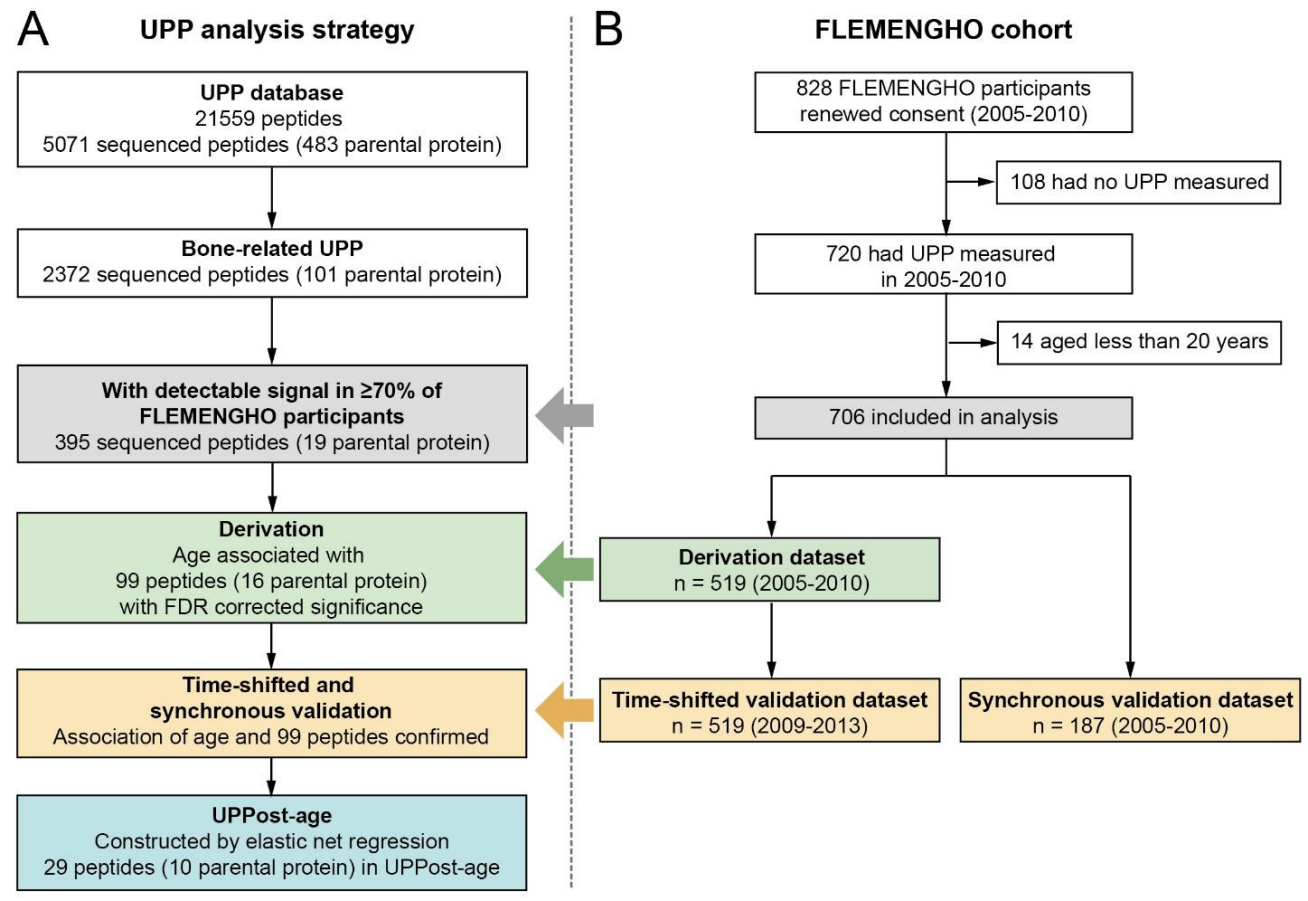
**Flow chart depicting the analysis strategy and the selection of FLEMENGHO participants Bone-related UPP refers to 2372 of 5071 sequenced urinary peptides derived from proteins implicated in skeleton development, bone formation and mineralization, ossification and other bone-related mechanism and metabolic processes (https://www.uniprot.org)**. Abbreviations: FLEMENGHO: Flemish Study on Environment, Genes, and Health Outcomes; UPP: urinary peptidomic profile; UPPost-age: age as predicted by UPP; FRD: false discovery rate.

From May 30, 2005, until May 31, 2010, participants were invited to a follow-up examination if their last known address was within 15 km of the local examination center (Eksel, Belgium) and if they had not withdrawn consent in any of the previous examination cycles (1985-2004). All participants who had a follow-up visit from 2005 to 2010 were invited to an additional follow-up examination from October 28, 2009, until March 19, 2013. Among 828 participants who renewed consent (Fig. 1), 122 were excluded from analysis, if they had not undergone UPP profiling (n = 108), or were aged younger than 20 years (n = 14). Therefore, three datasets were constructed including 706 participants (Fig. 1). The participants, who took part in both 2005-2010 and 2009-2013 follow-up examinations constituted the derivation dataset (n = 519), which included the 2005-2010 data, and the time-shifted validation dataset (n = 519), which included the 2009-2013 follow-up data. The remaining participants, who only took part in the 2005-2010 examination, constituted the synchronous validation dataset (n = 187).

### Clinical and biochemical variables

At each examination, study nurses administered a standardized questionnaire about each participant’s medical history, smoking and drinking habits, intake of medications and risk factors. Body height was measured with participants barefoot and standing firm against a wall. Arm span, measured by a flexible meter, was the distance from the tip of the middle finger from the left hand to the tip of the middle finger from the right hand with the both arms abducted at 90°. Body height and arm span were determined to the nearest centimeter. Urine samples for UPP analysis were aliquots of a 24-h urine sample collected within one week of the examination and deep frozen at -40 C. Venous blood samples were obtained after 8 hours of fasting. After centrifugation and aliquoting, blood-derived specimens were stored at -80°C. A single certified laboratory assessed the routine biochemistry, using quality controlled automated methods. The estimated glomerular filtration rate (eGFR) was computed from serum creatinine [13], measured by a modification of Jaffe’s methods with isotope-dilution calibration [14]. According to the Chronic Kidney Disease Epidemiology Collaboration equation [14], eGFR = 141 × minimum (Scrt/κ, 1)^α^ × maximum (Scrt/κ,1)^-1.209^ × 0.993^age^ × 1.018 (if female), where Scrt is the serum creatinine concentration in μmol/L, κ is 61.9 for women and 79.6 for men, and α is -0.329 for women and -0.411 for men; minimum indicates the minimum of Scrt/κ or 1 and maximum indicates the maximum of Scrt/κ or 1. Desphospho-uncarboxylated matrix Gla protein (dp-ucMGP), a biomarker reflecting vitamin K status [15], was measured on citrated plasma by ELISA.

Among 357 women, 35 (9.80%) had bone mineral density (BMD) assessed in an earlier examination cycle. Bone mineral density (BMD) was measured at the forearm just above the wrist by single photon absorptiometry (ND1100 bone density scanner; Nuclear Data Inc, Schaumburg, IL, USA). Distal scans of adult forearms traverse a mean of 35% trabecular bone, whereas in proximal scans this proportion declines to nearly 5 % [16]. Low BMD was defined as the distal and proximal bone density corrected for fat and bone width lower than the 40th percentile.

### Outcomes

Body-height-to-arm-span-ratio (HASR) is an anthropometric parameter reflecting the osteoporotic compression of the vertebrae [17,18]. Body height and arm span are approximately equal in young adults. With arm span as reference unaffected by aging, HASR reflects the age-related loss of body height. In 656 FLEMENGHO participants (50.6% women; median age at the first measurement: 40.5 year), examined twice in the interval from 1985 until 2008, height change over a median of 9.6 years (IQR: 6.5-15.5 years, range: 0.74-23.4 years), averaged 0.46 cm (SD: 1.51) [19] and was significantly correlated with HASR (r = -0.17, p < 0.0001; Supplementary Fig. 1). The sex-specific correlation coefficients were -0.24 in women (red circles) and -0.095 in men (green squares), respectively (*p* = 0.057 for the sex difference). Therefore, HASR was analyzed as a continuous outcome reflecting osteoporosis and the absolute difference of body height minus arm span dichotomized by the ≤ -3 cm threshold [17,18] as binary outcome.

At annual intervals, the vital status of FLEMENGHO participants was ascertained by record linkage with the National Population Registry in Brussels, Belgium. For all participants, data on the incidence of nonfatal outcomes were collected by a standardized questionnaire at follow-up visits, via structured telephone interviews, and by searching participants’ medical records at the four regional hospitals and University Hospitals Leuven, all serving the catchment area. The outcomes of interest were osteoporosis (ICD8 code 723.0; nonaccidental fractures, invasive osteosynthesis, spine stabilizing surgery, or initiation of bisphosphonate treatment). The history of fracture was blindly and independently reviewed by two researchers against the source data to exclude the accidental cases not attributable to osteoporosis. For analysis of the incidence of osteoporosis and osteoporotic fracture, the date of urine collection in 2005-2010 served as the baseline. For all outcomes, participants were censored at occurrence of the first event or on June 30, 2019, if no event had occurred.

### Urinary peptidomics

The methods for urine sample preparation, capillary electrophoresis coupled with mass spectrometry (CE-MS), peptide sequencing, calibration, and quality control of the mass spectrometric data have been published [20,21,22] and are described in the Supplementary Data (pages 2-4). By identifying the function of the parental protein (Supplementary Table 1) from the UniProt database (https://www.uniprot.org), we analyzed peptides derived from proteins implicated in skeleton development, bone formation and mineralization, ossification and other bone-related mechanism and metabolic processes. Among the bone-related sequenced urinary peptides, those with a detectable signal in ≥70% of study participants (Fig. 1 and Supplementary Table 1), which is a common approach in UPP analysis [23,24], were carried through to further analysis. This selection procedure resulted in a short list of 395 of 5071 sequenced peptides originating from 19 parental proteins of interest, of which the biological function is highlighted in Supplementary Table 1.

### Statistical analysis

Database management and statistical analysis were done using SAS, version 9.4 (maintenance level 5). The distributions of UPP were rank normalized, by sorting measurements from the smallest to the highest value and then applying the inverse cumulative normal function [25]. Within-group changes of continuous and categorical variables were assessed by the t statistic and McNemar’s test, respectively. Between-group means and proportions were compared using the large-sample z test and Fisher’s exact test. Significance was a *p*-value of 0.05, with adjustment for multiple testing if indicated.

The analysis was done according to predefined steps (Fig. 1). First, the association between chronological age and the selected 395 urinary peptides was investigated in the 2005-2010 derivation dataset (*n* = 519), with cumulative adjustments applied for the clustering within families (random effect), sex, body mass index, eGFR, current smoking, γ-glutamyltransferase (index of alcohol intake), fasting plasma glucose, physical activity, use of diuretics (yes *vs* no) [26], and socioeconomic status (high or middle *vs* low; 1 *vs* 0). Physical activity and socioeconomic status were derived by questionnaire as described previously [27]. After applying Benjamini-Hochberg false discovery rate (FDR) to adjust significance, 99 urinary peptides associated with chronological age were retained in further analyses.

Because the prevalence of osteoporosis differs considerably between women and men, the association between the urinary peptide levels and sex was investigated in the 2005-2010 derivation dataset (*n* = 519) by general linear models, first unadjusted and next adjusted as described above, but omitting the sex adjustment. Volcano plots were then constructed to evaluate the consistency of sex-specific UPP distributions among the derivation, time-shifted validation and synchronous validation datasets. To develop UPPost-age, the 99 peptides were subjected to elastic net regression. The L1 and L2 regression penalties were determined by 10-fold random cross-validation. This procedure was bootstrapped 1000 times to obtain the final estimates of L1 and L2 and the 95% confidence interval of the regression coefficients, linking each protein fragment with chronological age. Next, to validate the aging biomarker, the UPPost-age biomarker was evaluated in the time-shifted validation and synchronous validation datasets. Linear regression, logistic regression and Cox regression models were applied to assess the associations of HASR, HASD ≤ -3 cm (0, 1), and incident osteoporosis (0, 1) and osteoporotic fracture (0,1), with chronological age and UPPost-age. In a sensitivity analysis, the risk of osteoporosis was assessed by the median of plasma dp-ucMGP in view of the risk modulation in a previous air pollution study [28].

**Table 1:** Characteristics of FLEMENGHO participants.

Characteristic	Derivation dataset (n = 519)	Time-shifted validation dataset (n = 519)	*p*-value	Synchronous validation dataset (n = 187)	*p*-value
**Anthropometrics**					
Women (%)	261 (50.3)	261 (50.3)	…	96 (51.3)	0.13
Natural menopause (% of women)	121 (43.4)	148 (56.7)	0.056	44 (45.8)	0.95
Surgical menopause (% of women)	13 (5.0)	15 (5.7)	0.70	5 (5.2)	0.90
Men (%)	258 (49.7)	258 (49.7)	…	91 (48.7)	0.81
Age (years)	49.8 ± 14.4	54.6 ± 14.3	<0.0001	51.8 ± 16.4	0.13
Body weight (kg)	76.0 ± 14.7	78.2 ± 15.0	0.019	75.9 ± 15.3	0.91
Body height (cm)	169.3 ± 9.38	169.3 ± 9.48	0.98	168.3 ± 9.19	0.20
Arm span (cm)	171.0 ± 11.0	170.7 ± 10.9	0.67	170.1 ± 11.1	0.37
Body mass index (kg/m^2^)	26.5 ± 4.34	27.2 ± 4.34	0.0054	26.7 ± 4.29	0.57
HASR	0.99 ± 0.03	0.99 ± 0.03	0.39	0.99 ± 0.03	0.67
**Biochemical measurements**					
Serum creatinine (mg/dL)	86.1 ± 15.6	89.5 ± 21.3	0.0041	86.6 ± 13.7	0.69
Total serum calcium (mg/dL)	9.56 ± 0.44	9.72 ± 0.40	<0.0001	9.52 ± 0.44	0.85
Fasting plasma glucose (mg/dL)	88.7 ± 13.7	89.1 ± 14.4	0.71	89.8 ± 15.7	0.36
eGFR (mL/min/1.73m^2^)	81.2 ± 14.8	76.0 ± 15.2	<0.0001	79.6 ± 16.0	0.21
dp-ucMGP (mg/L)	4.35 (2.98, 6.01)	…	…	4.89 (3.03, 6.41)	0.20
γ-glutamyltransferase (U/L)	22.0 (15.0, 33.0)	22.0 (16.0, 33.0)	0.47	23.0 (14.0, 33.0)	0.96
FSH in women (U/L)	18.5 (4.00, 52.9)	40.2 (5.70, 63.3)	<0.0001	23.0 (4.75, 53.2)	0.66
**Life style**					
Physical activity (kcal/day)	1768 (1322, 2218)	1682 (1330, 2163)	0.18	1863 (1389, 2261)	0.45
Current smoking (%)	96 (18.5)	75 (14.5)	0.079	41 (21.9)	0.31
Current alcohol intake (%)	148 (28.5)	156 (30.1)	0.59	68 (36.4)	0.046
Middle or high socioeconomic status (%)	194 (37.4)	192 (37.0)	0.90	56 (29.9)	0.068
**Comorbidities**					
Diabetes mellitus (%)	20 (3.9)	35 (6.7)	0.38	6 (3.2)	0.69
Hypertension (%)	226 (43.5)	269 (51.8)	0.012	79 (42.2)	0.43
**Use of medications**					
Diuretics (%)	47 (9.1)	62 (12.0)	0.16	22 (11.8)	0.31
Contraceptive pill intake (% of women)	54 (20.7)	45 (17.2)	0.32	24 (25.0)	0.38
Hormonal substitution (% of women)	13 (5.0)	13 (5.0)	…	6 (6.2)	0.64

Abbreviations: eGFR: glomerular filtration rate derived from serum creatinine, using the Chronic Kidney Disease Epidemiology Collaboration equation; dp-ucMGP: desphospho-uncarboxylated matrix Gla protein; HASR: body-height-to-arm-span ratio; FSH: follicle-stimulating hormone. Data are n (%), mean ± SD, or median (IQR); p-values denote the significance of the difference between the derivation dataset and the validation datasets. Diabetes was as fasting plasma glucose of ≥126 mg/dL (≥7.0 mmol/L), a self-reported diagnosis, diabetes documented in practice or hospital records, or use of antidiabetic drugs. Hypertension was a blood pressure of ≥140 mm Hg systolic or ≥90 mm Hg diastolic or use of antihypertensive drugs. To convert serum creatinine from mg/dL to μmol/L, multiply by 88.42; total serum calcium from mg/dL to mmol/L, multiply by 0.25; serum cholesterol from mg/dL to mmol/L, multiply by 0.0259; glucose from mg/dL to mmol/L, multiply by 0.0555, dp-ucMGP from μg/L to pmol/L, multiply by 94.299.

## RESULTS

### Characteristics of participants

The derivation dataset and the time-shifted validation dataset (*n* = 519, Table 1) included 261 women (50.3%) and 258 men (49.7%). Age averaged 49.8 (SD: 14.4 years) and 54.6 (14.3 years) at the 2005-2010 and 2009-2013 examinations, respectively. At baseline, mean body height and arm span were 169.3 (9.38 cm) and 171.0 (11.0 cm), and their corresponding ratio, HASR, was 0.99 (0.03). Overall, 47 (9.1%) participants were on diuretics in the derivation dataset and 54 of 261 (20.7%) women were taking contraceptive pills and 13 (5.0%) were on hormonal substitution therapy. The characteristics of participants included in the derivation dataset (*n* = 519) and synchronous validation dataset (*n* = 187) were largely similar (*p* ≥ 0.046; Table 1). From the 2005-2010 to the 2009-2013 examinations, mean body mass index increased by +0.75 kg/m^2^ (SE: 0.27; *p* = 0.0054), mean eGFR declined by -5.20 mL/min/1.73m^2^ (SE: 0.93; *p* < 0.0001), while the prevalence of hypertension (from 43.5% to 51.8%; *p* = 0.012) and the proportion of women reaching menopause (from 43.4% to 56.7%; *p* = 0.056) increased.

### Associations with chronological age

With multivariable adjustment and correction for multiple testing applied, 99 sequenced peptides, derived from 16 parental proteins, were significantly associated with chronological age (Supplementary Table 2). For peptides derived from the same parental protein, these associations were directionally similar in most but not all instances (Supplementary Table 2). Associations were positive for IGF2 (insulin-like growth factor II; same directionality in 2 of 2 peptides) and MGP (matrix Gla protein; 2 of 2). For peptides derived from collagens the associations were predominantly negative: COL1A1 (33 of 38), COL1A2 (13 of 14), COL2A1 (6 of 10), COL3A1 (17 of 21) and COL6A1 (3 of 3). For peptides with the highest significance derived from the same parental protein in the derivation dataset, the consistency of the associations was confirmed in the time-shifted validation and synchronous validation datasets (Supplementary Table 3), based on the FDR-corrected *p* value in a 1-sided test (given the prior probability established by the significance in the derivation dataset). Of the 16 parental proteins, 9 (56.3%) and 10 (62.5%) were confirmed in the time-shifted validation or the synchronous validation datasets, respectively. Only COL11A1 and COL11A2 remained unconfirmed in either validation dataset (Supplementary Table 3 and Supplementary Table 4).

**Figure 2.**
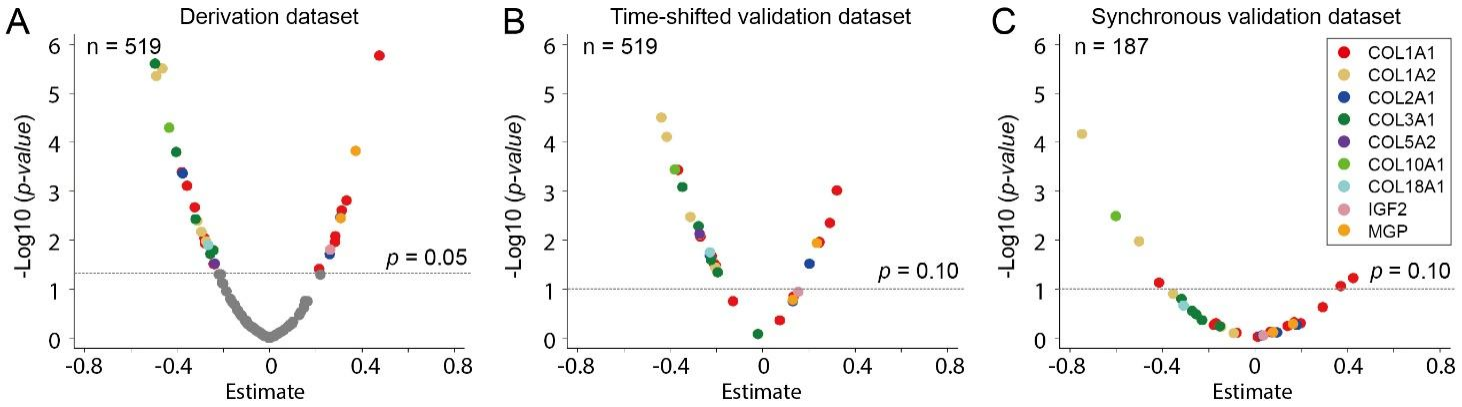
**Volcano plots depicting the sex-specific expression of bone-related urinary proteomics in the derivation (**A**), time-shifted validation (**B**) and synchronous validation (**C**) datasets**. Estimate was the association size of difference in urinary peptide level in women compared to men (men as reference) derived from the rank-normalized data in the adjusted general linear models. Adjusted models accounted for the clustering within families, age, body mass index, the glomerular filtration rate calculated from serum creatinine by the Chronic Kidney Disease Epidemiology Collaboration equation, current smoking, γ-glutamyltransferase as index of alcohol intake, fasting plasma glucose, physical activity, use of diuretics (yes *vs* no), and middle or high socioeconomic status (yes *vs* no). The 99 urinary peptides associated with chronological age were included in the analysis in the derivation dataset, and only those reaching significance (*p* < 0.05, colored dots; refer to Table S5) were included in the analysis of the time-shifted and synchronous validation datasets. The insignificant urinary peptides (gray dots in A) were not included in the replication analyses. *p*-values were corrected for the Benjamini-Hochberg false discovery rate (*p* < 0.10 in the validation datasets, given the prior probability established by the significance in the derivation dataset). 32 urinary peptides reached significance and identified 9 parental proteins in the adjusted models.

The sex-specific association of the 99 bone-related sequenced urinary peptides with chronological age was further explored, using multivariable-adjusted linear regression analysis. The peptide levels derived from IGF2 and MGP were significantly upregulated in women compared to men, whereas COL1A2, COL3A1, COL5A2, COL10A1 and COL18A1 were significantly down-regulated (Fig. 2 and Supplementary Table 5). Volcano plots demonstrated the consistency of these sex-specific associations with chronological age in the derivation and in the time-shifted validation and synchronous validation datasets (Fig. 2).

### Associations with UPPost-age

**Figure 3.**
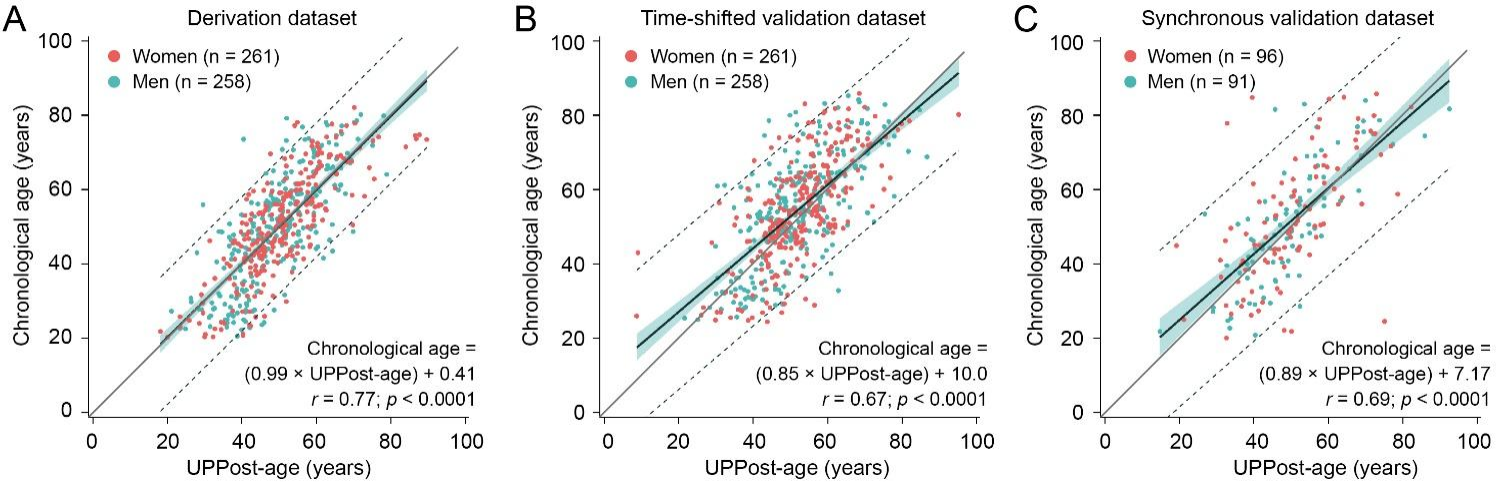
**Correlations between chronological age and the UPPost-age predicted by bone-related urinary proteome in FLEMENGHO participants**. In the derivation (**A**), time-shifted validation (**B**) and synchronous validation (**C**) datasets, the regression lines (solid black) are given with 95% confidence intervals for the predicted mean of chronological age (green band) and the predicted chronological age of individual participants (dotted lines). The grey line in the correlation plots is the identity line. Red and green dots represent women and men, respectively. The correlation coefficients (*r*), the significance (*p*) and the linear regression equation are given for each dataset. Abbreviations: FLEMENGHO: Flemish Study on Environment Genes, and Health Outcomes; UPP: urinary peptidomic profile; UPPost-age: age as predicted by UPP.

Elastic net regression retained 29 of 99 (29.3%) of the bone-related sequenced urinary peptides in the UPPost-age prediction model (Supplementary Table 6). The selected peptides originated from 10 parental proteins, mainly collagen fragments, IGF2 and MGP (Supplementary Table 6). The UPPost-age was similar to the chronological age in the derivation set (49.8 years [SE: 0.49] *vs* 49.8 years [0.63]; *p* = 0.98) and synchronous validation set (50.3 years [0.94] *vs* 51.8 years [1.20]; *p* = 0.088), whereas UPPost-age was lower than chronological age in the time-shifted validation dataset (52.1 years [0.50] *vs* 54.6 years [0.63]; *p* < 0.0001). Compared to the derivation set, the regression slopes were similar in the synchronous validation dataset (*p* = 0.17; Fig. 3), but slightly smaller in time-shifted validation dataset (*p* = 0.012; Fig. 3). For a 10-years increase in the UPPost-age (Fig. 3), the chronological age increased by 9.9 years (95% CI: 9.2-10.6 years; *r* = 0.77) in the derivation datasets, 8.5 years (95% CI: 7.7-9.4 years; *r* = 0.67) in the time-shifted validation dataset, and 8.9 years (95% CI: 7.5-10.2 years; *r* = 0.69) in the synchronous validation dataset. Among the 35 women who had both BMD and UPP measured, UPPost-age was significantly higher in those with low BMD (59.6 years [SD: 7.12] *vs* 54.0 years [6.68], *p* = 0.047).

### Association of HASD and HASR with age

In a cross-sectional analysis of the FLEMENGHO baseline data (2005-2010), the risk of having HASD ≤ -3 cm, was related to age and UPPost-age (Supplementary Table 7). The odds ratios expressing the risk of having HASD ≤ -3 cm per 10-year increment were significant for age in all participants, men, women and postmenopausal women (p ≤ 0.025). For UPPost-age, significance was maintained in all participants and in women with odds ratios amounting to 1.31 (95% CI: 1.12, 1.53; p < 0.0007) and 1.43 (1.14, 1.81; p = 0.0024), respectively (Supplementary Table 7).

The 706 participants making up the FLEMENGHO study population included 108 unrelated participants and 598 related participants from 16 single-generation families and 58 multigeneration pedigrees. In multivariable-adjusted regression analyses, HASR was significantly associated with chronological age (*p* ≤ 0.001) and UPPost-age (*p* ≤ 0.011) in all FLEMENGHO participants, men and women, with a similar trend in postmenopausal women. When the population was dichotomized by the -3 cm HASD threshold, the directionality of the associations was maintained, but significance was lost in women and postmenopausal women (Table 2).

### Incidence of osteoporosis

During a median follow-up interval of 4.89 years (IQR: 4.44 - 5.19 years), 53 participants developed osteoporosis, of whom 16 had an osteoporosis diagnosis in practice or hospital files and 37 experienced an osteoporotic fracture. With adjustments applied for clustering within families and sex, in all participants, the risk of osteoporosis (53 cases) expressed per 10-year increment in age and UPPost-age, increased by 21% and 36% (*p* ≤ 0.044), respectively. In women, the corresponding estimates adjusted for clustering within families were 55% and 60% for incident osteoporosis (37 cases; *p* ≤ 0.0003) and 42% and 44% for osteoporotic fractures (25 cases; *p* ≤ 0.017). Full adjustment for all risk factors for osteoporosis removed the significance of the associations in Table 3.

Independent of chronological age (Supplementary Fig. 2), serum dp-ucMGP was correlated with UPPost-age (*r* = 0.41; *p* < 0.0001). Dichotomized by the median of dp-ucMGP (≥ 4.50 and < 4.50 mg/L), the risk of osteoporosis was significantly associated with UPPost-age in the higher dp-ucMGP category (HR: 1.35 [1.01, 1.80]; *p* = 0.040), but not in the lower category (HR: 1.31 [0.84, 2.05]; *p* = 0.24).

**Table 2:** Body-height-to-arm-span ratio in relation to baseline age in FLEMENGHO participants (2005-2010).

HASR (× 10^-3^) Group analyzed	*n*	Chronological age		UPPost-age	
		Estimate (95% CI)	*p*-value	Estimate (95% CI)	*p*-value
**All participants**	706	-4.84 (-6.32, -3.36)	<0.0001	-4.67 (-6.47, -2.87)	<0.0001
HASD > -3 cm	428	-1.85 (-3.26, -0.43)	0.011	-2.22 (-3.89, -0.55)	0.0093
HASD ≤ -3 cm	278	-1.84 (-3.34, -0.34)	0.016	-2.39 (-4.18, -0.59)	0.0095
**Men**	349	-5.39 (-7.41, -3.36)	<0.0001	-4.31 (-6.90, -1.73)	0.0011
HASD > -3 cm	161	-2.84 (-5.00, -0.67)	0.010	-2.60 (-5.27, 0.07)	0.056
HASD ≤ -3 cm	188	-1.45 (-3.26, 0.36)	0.12	-2.40 (-4.60, -0.20)	0.032
**Women**	357	-4.51 (-6.77, -2.25)	0.0001	-4.86 (-7.40, -2.32)	0.0002
HASD > -3 cm	267	-1.01 (-2.95, 0.93)	0.30	-1.79 (-3.95, 0.36)	0.10
HASD ≤ -3 cm	90	-2.40 (-5.46, 0.66)	0.12	-2.01 (-5.39, 1.37)	0.24
**Menopausal women**	183	-4.91 (-10.1, 0.26)	0.063	-3.67 (-8.22, 0.87)	0.11
HASD > -3 cm	121	-1.07 (-5.35, 3.20)	0.62	-3.24 (-6.94, 0.47)	0.086
HASD ≤ -3 cm	62	0.36 (-5.64, 6.36)	0.90	1.28 (-3.86, 6.43)	0.62

Abbreviations; HASD: the difference of body height minus arm span; HASR: body-height-to-arm-span ratio; n: the number of participants in the category; UPPost-age: age as predicted by the urinary proteomic profile; 95% CI: 95% confidence interval. Estimates were derived by linear regression analysis with HASR as the continuous outcome in all participants and in two subgroups dichotomized by the HASD threshold (> -3 vs ≤ -3 cm). Estimates, given with 95% CI, express the association size of HASR per 10-year increment in chronological age or UPPost-age. Estimates are partial regression coefficients adjusted for clustering within families, sex (in all participants only), current smoking, γ-glutamyltransferase, fasting plasma glucose, physical activity, use of diuretics (yes vs no), and socioeconomic status (high or middle vs low). To increase readability, leading zeros were removed by multiplying the estimates by 10^3^; the actual values can be obtained by multiplying the estimates by 10^-3^.

### DISCUSSION

We constructed a novel aging-related biomarker indicative of osteoporosis, UPPost-age, and validated UPPost-age cross-sectionally and longitudinally in the family-based FLEMENGHO study, which is representative for Europeans living in northern Belgium. Four lines of evidence support this statement. First, chronological age was associated with bone-related UPP fragments, including collagens, IGF2 and MGP in the derivation and validation datasets. Second, among the 99 bone-related urinary peptides associated with chronological age, 32 (32.3%) showed a sex-specific distribution probably explaining sexual heterogeneity in the molecular pathogenic mechanisms in osteoporosis. Third, HASR and HASD ≤ -3 cm, anthropometric measurements reflecting osteoporosis, were associated with UPPost-age. Finally, the association between osteoporosis and UPPost-age reached significance in all participants and in women and osteoporotic fracture was associated with UPPost-age in women. In line with the aforementioned results, there was a positive correlation between the plasma levels of dp-ucMGP and UPPost-age and the risk of osteoporosis was higher in participants with high plasma dp-ucMGP, a marker of vitamin K deficiency [15]. Its activation requires a vitamin K-dependent γ-glutamate carboxylation, which is also needed for key proteins maintaining the integrity of bone tissue, including osteocalcin, GAS6, periostin and protein S [29], which play a key role in the differentiation and gene expression in osteoblasts and in the mineralization of the bone extracellular matrix [30].

**Table 3:** Risk of osteoporosis in relation to age in FLEMENGHO participants.

Variable	n/N	Chronological age		UPPost-age	
		HR (95% CI)	*p*-value	HR (95% CI)	*p*-value
**All participants**					
Osteoporosis	53/706	1.21 (1.00, 1.46)	0.044	1.36 (1.09, 1.68)	0.0057
Osteoporotic fracture	37/706	1.05 (0.84, 1.31)	0.66	1.17 (0.90, 1.53)	0.24
**Men**					
**Osteoporosis**	16/349	0.72 (0.51, 1.01)	0.058	0.81 (0.52, 1.26)	0.36
**Osteoporotic fracture**	12/349	0.56 (0.36, 0.86)	0.0083	0.66 (0.39, 1.12)	0.12
**Women**					
**Osteoporosis**	37/357	1.55 (1.23, 1.95)	0.0003	1.60 (1.25, 2.03)	0.0001
**Osteoporotic fracture**	25/357	1.42 (1.07, 1.88)	0.014	1.44 (1.07, 1.94)	0.017
**Menopausal women**					
**Osteoporosis**	32/183	1.17 (0.82, 1.67)	0.39	1.31 (0.96, 1.78)	0.087
**Osteoporotic fracture**	20/183	1.13 (0.72, 1.77)	0.59	1.22 (0.82, 1.81)	0.32

Abbreviations: n/N: number of incident cases/number at risk; UPP: urinary peptidomic profile; UPPost-age: age as predicted by the urinary proteomic profile; HR: hazard ratio; 95% CI: 95% confidence interval. Of 53 incident osteoporosis cases, 16 had the diagnosis documented in practice or hospital records and 37 experienced an osteoporotic fracture. HRs, given with 95% CI, express the relative risk per 10-year increment in chronological age or UPPost-age. The HRs were adjusted for clustering within families and in all participants additionally for sex.

The current findings are consistent with previous FLEMENGHO publication [9], showing that a general aging UPP biomarker without focus on bone tissue proteins (UPPost-age) was associated with osteoporosis and fracture risk in all participants and women. However, UPPost-age only had 8 of 29 (27.6%) urinary peptide segment and 3 of 10 (30.0%) parental proteins in common with UPP-age. Furthermore, a case-control study identified a distinctive serum proteomics profile, which differed between healthy controls compared with patients with osteopenia and osteoporosis (Q^2^ = 0.7295, R^2^ = 0.9180). Most of the dysregulated proteins were immunoglobins, complement proteins and cytoskeletal proteins [31]. Focusing on in vitro experiments, a comparative analysis of proteins expressed by three osteoblast-derived extracellular matrix preparations identified more than 1000 proteins with differing levels, including those specifically present in mineralized matrix, such as growth factors and proteins linked to cell migration and angiogenesis [32].

Type-I collagen is the most abundant constituents of the organic extracellular matrix in bones tissue, accounting for 90% of total collagen [33,34]. The main function of collagens is to provide mechanical support and to act as a scaffold for bone cells. COL1A1 participates in various physiological processes, including bone trabecular formation, ossification, osteoblast differentiation and skeletal system development, while COL1A2 is involved in bone mineralization (Supplementary Table 1). In the type-I collagen degradation, small fragments are first released by collagenases, whereas larger fragments are further degraded by gelatinases with the resulting smaller fragments being further cleaved by gelatinases [35]. Only breakdown products of mature collagen appear in urine [35]. Current findings showed an inverse association between chronological age and type-I collagen fragments (Supplementary Table 2), indicating that the level of collagen I fragments decreased in urine with advancing age. Furthermore, in the current study, chronological age was negatively associated with the urinary levels of peptide fragments derived from COL2A1, COL3A1 and COL6A1 (Supplementary Table 2). COL2A1 participate in cartilage development and endochondral bone morphogenesis [36]. COL3A1 serves as the stimulator of cell growth and messenger RNA expression in normal osteoblasts and bone marrow mesenchymal stem cells [37]. COL6A1 deficiency alters the configuration and shape of osteoblasts, which affects the maintenance of bone mass [38,39]. The inverse association of urine peptide fragments derived from these collagens with age might therefore reflect the reduced expression of these functional collagens with age.

The present study demonstrated that the bone-related UPP displayed a sex-specific pattern with women having lower levels than men of urinary peptides derived from collagens, IGF2 and MGP (Fig. 3). The possible explanation for the up-regulated IGF2 in women might be an upregulation for bone repair and remodeling [40]. Along similar lines, a previous study [41] reported the association of 90 urinary peptides with sex and verified these findings in a general population sample (n = 337) and in patients with type-2 diabetes (*n* = 1442). These peptides were derived from 29 parental proteins, including COL1A1, COL1A2, COL3A1, COL10A1, COL18A1 and PIGR [41]. Subgroup analysis in the healthy individuals below 45 years and over 55 years revealed that 51 and 66 urinary peptides were associated with sex, respectively, indicating that these sex-specific differences became more pronounced after menopause [41]. Osteoporosis is an aging-related skeletal disease with an exceedingly higher prevalence and high incidence of osteoporotic fractures in postmenopausal women, compared to men of similar age [42,43]. Among the urinary peptides retained in the UPPost-age prediction model, 10 (34.5%) displayed the sex-specific difference. Identification of sex-associated UPP differences might help to have a better understanding in pathogenic molecular mechanisms in women compared to men, irrespective of the disease of interest. Our current observations might prompt research into novel circulating or urinary biomarkers with high diagnostic accuracy over and beyond the levels of sex hormones and UPPost-age.

### Strong points and limitations

The strong points of the current study are the replication and validation of UPPost-age prediction model within the FLEMENGHO cohort, and the demonstration of its clinical relevance given its association with osteoporosis. Notwithstanding these strong points, the current study must be interpreted within the context of its limitations. First, the validation of UPPost-age prediction model was based on the HASR, HASD ≤ -3 cm and the incidence of osteoporosis. Not all the participants had BMD, the gold standard of osteoporosis diagnosis, which might have led to an underestimation of the incidence of clinically asymptomatic osteoporosis. However, UPPost-age was significantly higher in the women with low BMD in the study population. Furthermore, the incidence rates per 100 persons were 7.51% (95% CI: 5.56, 9.45%) for osteoporosis and 5.24% (3.60, 6.88%) for osteoporotic fracture during the long-term follow-up, which was comparable to previous studies [2,44]. Second, osteoporosis causes high health care expenditures, predominantly due to the treatment and clinical management of severe fractures [45]. A health economic analysis was not within the scope of the current study. Further health economic studies are needed to compare the cost of osteoporosis management with and without the application of UPPost-age as an aging biomarker indicating the risk of osteoporotic fractures in women and as actionable guide for and early intervention and prevention. Third, the FLEMENGHO cohort was recruited in a high-income country, where residents have ample access to calcium-rich food, such as milk and cheese, and nutrients, such as fermented cheese and green vegetables rich in vitamin K. Therefore, further validation of the UPPost-age prediction model is necessary in other ethnicities, less affluent countries and special populations, in particular among high risk postmenopausal women [46], organ transplantation recipients with immunotherapy [47] and patients with vitamin D deficiency [48]. Finally, the number of events in some strata in Table 3 was too low to allow a full adjustment. However, the cross-sectional analyses in Table 2, support the findings reported in Table 3 with partial adjustment. Independent replication in other cohorts might strengthen our current findings. Moreover, the results in Table 3 are in keeping with the higher osteoporosis risk in women compared with men.

### Conclusion

An aging-related UPP signature focused on peptide fragments derived from bone tissue-related proteins is associated with the risk of osteoporosis and osteoporotic fracture. The UPPost-age biomarker is registered as an in-vitro diagnostics in Germany and the European Union, providing patients, clinicians and trialists with a novel approach to predict the risk of osteoporosis at an early stage, when prevention of osteoporotic fractures is still possible.

## Supplementary Materials

The Supplementary data can be found online at: www.aginganddisease.org/EN/10.14336/AD.2024.0303.



## Data Availability

Anonymized participants’ data can be made available upon request directed to the corresponding author. Proposals will be reviewed with scientific merit and feasibility as the criteria. After approval of a proposal, data can be shared via a secure online platform after signing a data access and confidentiality agreement. Data will be made available for a maximum of 2 years after a data sharing agreement has been signed.
