# Supplementary material for: Osteoporosis in Relation to a Bone-Related Aging Biomarker Derived from the Urinary Proteomic Profile: A Population Study
Source: Aging Dis. 2024 Mar 3;16(1):633–44. doi: 10.14336/AD.2024.0303 (PMC11745457; doi:10.14336/AD.2024.0303)
Supplement: Supplementary file 1 [file AD-16-1-633-s.pdf]

# Osteoporosis in Relation to a Bone-Related Aging Biomarker Derived from the Urinary Proteomic Profile: A Population Study

**Yu-Ling Yu, Dries S. Martens, De-Wei An, Babangida Chori, Agnieszka Latosinska, Justyna Siwy, Augustine N. Odili, Katarzyna Stolarz-Skrzypek, Gladys E. Maestre, Kei Asayama, Yan Li, Peter Verhamme, Karel Allegaert, Harald Mischak, Tim S. Nawrot, Jan A. Staessen**

***Supplementary Data***

*Osteoporosis in Relation to a Bone-Related Aging Biomarker Derived From the Urinary Proteomic Profile: A Population Study*

Yu-Ling Yu, Dries S. Martens, De-Wei An, Babangida Chori, Agnieszka Latosinska, Justyna Siwy, Augustine N. Odili, Katarzyna Stolarz-Skrzypek, Gladys E. Maestre, Kei Asayama, Yan Li, Peter Verhamme, Karel Allegaert, Harald Mischak, Tim S. Nawrot, Jan A. Staessen

## Table of Contents

**Urinary Proteomics** p2

Sample preparation and CE-MS analysis p2

CE-MS data processing p3

Sequencing of peptides p4

References p4

**Table S1** Bone-related function of 19 parental protein identified by 395 sequenced urinary peptides p5

**Table S2** Association of 99 bone-related urinary peptides with chronological age in the derivation dataset p6

**Table S3** Association of chronological age with 16 bone-related urinary peptides p11

**Table S4** Number of replicated bone-related urinary peptides identifying the parental proteins p12

**Table S5** Sex difference of bone-related urinary peptides in the derivation dataset p13

**Table S6** Weights assigned by elastic net regression to the multidimensional UPP biomarker reflecting aging p15

**Table S7** Risk of having HASD ≤ 3 cm in FLEMENGHO participants at baseline (2005-2010) p17

**Figure S1** Correlation between height loss and body-height-to-arm-span ratio in 656 FLEMENGHO participants p18

**Figure S2** Regression plot of dp-ucMGP with chronological age and UPPost-age in 597 FLEMENGHO participants p19

**Urinary Proteomics**

***Sample preparation and CE-MS analysis***

As described in detail elsewhere [1], urine aliquots were thawed and 700 µl mixed with 700 µL of 2 M urea, 10 mM $NH_4OH$ containing 0.02 % SDS. Subsequently, samples were ultrafiltered using a Centristat 20 kDa cut-off centrifugal filter device (Satorius, Göttingen, Germany) to eliminate high molecular weight proteins. The obtained filtrate was desalted using a PD 10 gel filtration column (GE Healthcare Bio Sciences, Uppsala, Sweden) to remove urea, electrolytes and salts as well as to enrich the polypeptides. The samples were lyophilized and stored at 4°C before usage. Shortly before CE-MS analysis, the samples were re-suspended in 10 µL HPLC-grade $H_2O$. Samples were injected into CE-MS with 2 psi for 99 sec, resulting in injection volumes of ~280 nL.

A P/ACE MDQ capillary electrophoresis system (Beckman Coulter, Fullerton, CA) was coupled with a Micro-TOF MS (Bruker Daltronic, Bremen, Germany). A solution of 20% acetonitrile (Sigma-Aldrich, Taufkirchen, Germany) in HPLC-grade $H_2O$ (Roth, Karlsruhe, Germany) supplemented with 0.94% formic acid (Sigma-Aldrich) was used as running buffer. For CE-MS analysis, the electrospray ionization interface from Agilent Technologies (Palo Alto, CA) was set to a potential of -4.0 to -4.5 kV. Spectra were recorded over an *m*/*z* range of 350-3000 and accumulated every 3 s.

***CE-MS data processing***

After the CE-MS analysis, mass spectral ion peaks representing identical molecules at different charge states were deconvoluted into single masses using MosaFinder software [2]. Only signals with z>1 observed in a minimum of 3 consecutive spectra with a signal-to-noise ratio of at least 4 were considered. The resulting peak list characterizes each polypeptide by its mass and migration time. Data were calibrated utilizing 3151 internal standards as reference data points for mass and migration time by applying global and local linear regression, respectively as described previously. Reference signals of 29 abundant peptides were used as internal standards for calibration of signal intensity using local linear regression [3]. This procedure was shown to be an easy and reliable method to address both analytical and dilution variances and differences in glomerular filtration in a single calibration step. This procedure was shown to be highly reproducible and to address both analytical and dilution variances in a single calibration step [3]. The obtained peak list characterizes each polypeptide by its calibrated molecular mass (Da), calibrated CE migration time (min) and normalized signal intensity. All detected peptides were deposited, matched, and annotated in a Microsoft SQL database allowing further statistical analysis.

***Sequencing of peptides***

Candidate biomarkers were sequenced using CE-MS/MS or LC-MS/MS analysis, as described in detail [4]. MS/MS experiments were using an Ultimate 3000 nano-flow system (Dionex/LC Packings, Thermo Fisher Scientific Inc., Waltham, MA) or a P/ACE MDQ capillary electrophoresis system (Beckman Coulter, Fullerton, CA), both connected to an LTQ Orbitrap hybrid mass spectrometer (Thermo Fisher Scientific GmbH, Bremen, Germany) equipped with a nano-electrospray ion source. The mass spectrometer was operated in data-dependent mode to automatically switch between MS and MS/MS acquisition. Survey full-scan MS spectra (from *m*/*z* 300–2,000) were acquired in the Orbitrap. Ions were sequentially isolated for fragmentation. Data files were searched against the

UniProt human nonredundant database, using Proteome Discoverer 2.4 and the SEQUEST search engine. Relevant settings were: no fixed modifications, oxidation of methionine and proline as variable modifications. The minimum precursor mass was set to 790 Da, maximum precursor mass to 6000 Da with a minimum peak count of 10. The high-confidence peptides were defined by cross-correlation (Xcorr) >1.9 and rank = 1. Precursor mass tolerance and fragment mass tolerance were 5 ppm and 0.05 Da, respectively. For further validation of obtained peptide sequences, the correlation between peptide charge at the working pH of 2 and CE-migration time was utilized to minimize incorrect sequence assignment [5]. Calculated CE-migration time of the sequence candidate based on its peptide sequence (number of basic amino acids) was compared to the experimental migration time.

**References**


1. Mischak H, Vlahou A, Ioannidis JPA (2013). Technical aspects and inter-laboratory variability in native peptide profiling: the CE-MS experience. Clin Biochem, 46: 432-43.
2. Latosinska A, Siwy J, Mischak H, Frantzi M (2019). Peptidomics and proteomics based on CE-MS as a robust tool in clinical application: the past, the present, and the future. Electrophoresis, 40: 2294-308.
3. Jantos-Siwy J, Schiffer E, Brand K, Schumann G, Rossing K, Delles C, et al. (2009). Quantitative urinary proteome analysis for biomarker evaluation in chronic kidney disease. J Proteome Res, 8: 268-81.
4. Klein J, Papadopoulos T, Mischak H, Mullen W (2014). Comparison of CE-MS/MS and LC-MS/MS sequencing demonstrates significant complementarity in natural peptide identification in human urine. Electrophoresis, 35: 1060-4.
5. Zürbig P, Renfrow MB, Schiffer E, Novak J, Walden M, Wittke S, et al. (2006). Biomarker discovery by CE-MS enables sequence analysis via MS/MS with platform-independent separation. Electrophoresis, 27: 2111-25.

**Supplementary Table 1.** Bone-related function of 19 parental protein identified by 395 sequenced urinary peptides.

| Parental proteins identified from the amino-acid sequence | | | Number of peptides in analysis | Bone-related function |
|---|---|---|---|---|
| **Accession number** | **Symbol** | **Name** | | |
| P02452 | COL1A1 | Collagen alpha-1(I) chain | 199 | Bone trabecula formation, cartilage development involved in endochondral bone morphogenesis, embryonic skeletal system development, endochondral ossification, extracellular matrix structural constituent and organization, ossification, osteoblast differentiation, skeletal system development |
| P08123 | COL1A2 | Collagen alpha-2(I) chain | 37 | Bone mineralization, extracellular matrix structural constituent and organization, skeletal system development |
| P02458 | COL2A1 | Collagen alpha-1(II) chain | 21 | Extracellular matrix structural constituent, cartilage condensation, cartilage development involved in endochondral bone morphogenesis, chondrocyte differentiation, embryonic skeletal joint morphogenesis, endochondral ossification, skeletal system development |
| P02461 | COL3A1 | Collagen alpha-1(III) chain | 94 | Extracellular matrix structural constituent and organization, limb joint morphogenesis |
| P53420 | COL4A4 | Collagen alpha-4(IV) chain | 3 | Extracellular matrix structural constituent, high expression in calvaria |
| P05997 | COL5A2 | Collagen alpha-2(V) chain | 5 | Extracellular matrix organization, ossification, skeletal system development |
| P12109 | COL6A1 | Collagen alpha-1(VI) chain | 5 | Extracellular matrix structural constituent, osteoblast differentiation |
| Q14055 | COL9A2 | Collagen alpha-2(IX) chain | 2 | Extracellular matrix structural constituent and organization, skeletal system development |
| Q14050 | COL9A3 | Collagen alpha-3(IX) chain | 2 | Extracellular matrix structural constituent and organization, involvement in multiple epiphyseal dysplasia |
| Q03692 | COL10A1 | Collagen alpha-1(X) chain | 1 | Extracellular matrix structural constituent and organization, skeletal system development |
| P12107 | COL11A1 | Collagen alpha-1(XI) chain | 7 | Extracellular matrix structural constituent and organization, cartilage condensation, embryonic skeletal system morphogenesis, ossification |
| P13942 | COL11A2 | Collagen alpha-2(XI) chain | 4 | Extracellular matrix structural constituent and organization, cartilage development, skeletal system development |
| Q5TAT6 | COL13A1 | Collagen alpha-1(XIII) chain | 4 | Extracellular matrix structural constituent and organization, endochondral ossification, skeletal system development |
| P39060 | COL18A1 | Collagen alpha-1(XVIII) chain | 2 | Extracellular matrix structural constituent and organization, collagen fibril organization, skeletal system development |
| Q14993 | COL19A1 | Collagen alpha-1(XIX) chain | 2 | Extracellular matrix structural constituent and organization, skeletal system development |
| Q96P44 | COL21A1 | Collagen alpha-1(XXI) chain | 1 | Collagen-containing extracellular matrix, expression in bone marrow |
| P01344 | IGF2 | Insulin-like growth factor II | 3 | Growth factor and hormone activity, osteoblast differentiation |
| P08493 | MGP | Matrix Gla protein | 2 | Structural constituent of bone, cartilage condensation, ossification, regulation of bone mineralization |
| Q86Y38 | XYLT1 | Xylosyltransferase 1 | 1 | Embryonic skeletal system development, ossification involved in bone maturation |

For analysis, 395 sequenced urinary peptides derived from 19 parental proteins with a detectable signal in ≥70% FLEMENGHO participants were selected (see Figure 1). Accession numbers refer to the Uniprot database (http://www.uniprot.org/uniprot), which substantiated the bone-related function of the parental proteins.

**Supplementary Table 2.** Association of 99 bone-related sequenced urinary peptides with chronological age in the derivation dataset (starts)

| Peptide ID number | Amino-acid sequence | Parental proteins identified from the amino-acid sequence | | | β (95% confidence interval) | *p*-value |
|---|---|---|---|---|---|---|
| | | Accession number | Symbol | Name | | |
| e99907132 | EpGSpGENGAPGQmGPR | P02452 | COL1A1 | Collagen alpha-1(I) chain | 0.281 (0.203, 0.359) | 9.21E-11 |
| e99910072 | GLpGTGGPpGENGKpGEpGPKG | P02461 | COL3A1 | Collagen alpha-1(III) chain | -0.232 (-0.309, -0.155) | 3.29E-08 |
| e99901132 | cDDYRLcE | P08493 | MGP | Matrix Gla protein | 0.230 (0.154, 0.305) | 3.85E-08 |
| e99906708 | pGpAGEKGSpGADGPAGAP | P02452 | COL1A1 | Collagen alpha-1(I) chain | 0.250 (0.169, 0.332) | 5.19E-08 |
| e99904196 | PSGDQGASGpAGPSGP | P02458 | COL2A1 | Collagen alpha-1(II) chain | 0.237 (0.156, 0.318) | 7.19E-08 |
| e99920065 | ARGNDGARGSDGQpGppGPPGTAGFPGSpGAKGEV GpAGSpGSNGApG | P02461 | COL3A1 | Collagen alpha-1(III) chain | -0.241 (-0.323, -0.159) | 7.27E-08 |
| e99912851 | LDGAKGDAGPAGPKGEpGSpGENGApG | P02452 | COL1A1 | Collagen alpha-1(I) chain | -0.227 (-0.304, -0.151) | 1.52E-07 |
| e99917856 | ARGNDGARGSDGQPGPpGppGTAGFpGSpGAKGEV GP | P02461 | COL3A1 | Collagen alpha-1(III) chain | -0.235 (-0.317, -0.153) | 1.78E-07 |
| e99919107 | DQGPVGRTGEVGAVGPpGFAGEKGpSGEAGTAGPP GTpGPQG | P08123 | COL1A2 | Collagen alpha-2(I) chain | -0.218 (-0.297, -0.139) | 3.00E-07 |
| e99917922 | NTGApGSPGVSGPKGDAGQpGEKGSpGAQGpPGAP GPLG | P02461 | COL3A1 | Collagen alpha-1(III) chain | -0.234 (-0.318, -0.151) | 3.04E-07 |
| e99916966 | PpGESGREGApGAEGSpGRDGSPGAKGDRGETGP | P02452 | COL1A1 | Collagen alpha-1(I) chain | -0.215 (-0.295, -0.135) | 3.23E-06 |
| e99906441 | TGSpGSpGPDGKTGPpGP | P02452 | COL1A1 | Collagen alpha-1(I) chain | -0.204 (-0.283, -0.126) | 7.75E-06 |
| e99912986 | ADGQpGAKGEpGDAGAKGDAGPPGPAGP | P02452 | COL1A1 | Collagen alpha-1(I) chain | -0.203 (-0.282, -0.125) | 9.27E-06 |
| e99901661 | DGpSGAEGpPGp | P02458 | COL2A1 | Collagen alpha-1(II) chain | 0.187 (0.107, 0.267) | 2.49E-05 |
| e99918573 | DQGPVGRTGEVGAVGpPGFAGEKGPSGEAGTAGPp GTpGP | P08123 | COL1A2 | Collagen alpha-2(I) chain | -0.183 (-0.263, -0.102) | 3.26E-05 |
| e99919745 | SKGESGNKGEpGSAGPQGPpGpSGEEGKRGPNGEA GSAGPPGPpG | P08123 | COL1A2 | Collagen alpha-2(I) chain | -0.187 (-0.270, -0.105) | 3.43E-05 |
| e99905560 | GSpGSpGPDGKTGPPGp | P02452 | COL1A1 | Collagen alpha-1(I) chain | -0.191 (-0.269, -0.113) | 3.61E-05 |
| e99900340 | cDDYRLc | P08493 | MGP | Matrix Gla protein | 0.189 (0.108, 0.270) | 4.30E-05 |
| e99909891 | SEGSPGHpGQPGpPGpPGApGP | P02461 | COL3A1 | Collagen alpha-1(III) chain | -0.189 (-0.271, -0.106) | 4.88E-05 |
| e99906709 | GSpGSpGPDGKTGPpGPAG | P02452 | COL1A1 | Collagen alpha-1(I) chain | -0.186 (-0.264, -0.108) | 5.84E-05 |
| e99906154 | EDGHpGKPGRpGERG | P08123 | COL1A2 | Collagen alpha-2(I) chain | -0.180 (-0.261, -0.098) | 6.07E-05 |
| e99912949 | LDGAKGDAGPAGpKGEpGSpGENGApG | P02452 | COL1A1 | Collagen alpha-1(I) chain | -0.191 (-0.271, -0.111) | 6.40E-05 |

**Supplementary Table 2** Association of 99 bone-related sequenced urinary peptides with chronological age in the derivation dataset (continued)

| Peptide ID number | Amino-acid sequence | Parental proteins identified from the amino-acid sequence | | | β (95% confidence interval) | *p*-value |
|---|---|---|---|---|---|---|
| | | Accession number | Symbol | Name | | |
| e99917311 | NTGApGSpGVSGpKGDAGQpGEKGSPGAQGPPGAPGp | P02461 | COL3A1 | Collagen alpha-1(III) chain | -0.186 (-0.270, -0.102) | 9.67E-05 |
| e99915778 | ESGREGAPGAEGSPGRDGSpGAKGDRGETGp | P02452 | COL1A1 | Collagen alpha-1(I) chain | -0.186 (-0.266, -0.106) | 0.00011 |
| e99911415 | IGPpGPAGApGDKGESGPSGPAGPTG | P02452 | COL1A1 | Collagen alpha-1(I) chain | -0.182 (-0.261, -0.104) | 0.00012 |
| e99903016 | ApGDRGEPGPpGP | P02452 | COL1A1 | Collagen alpha-1(I) chain | -0.187 (-0.268, -0.105) | 0.00016 |
| e99903372 | DGQPGAKGEpGDAG | P02452 | COL1A1 | Collagen alpha-1(I) chain | -0.184 (-0.266, -0.103) | 0.00017 |
| e99911777 | ANGApGNDGAKGDAGApGApGSQGAPG | P02452 | COL1A1 | Collagen alpha-1(I) chain | -0.188 (-0.271, -0.104) | 0.00019 |
| e99903120 | EGSpGRDGSpGAK | P02452 | COL1A1 | Collagen alpha-1(I) chain | 0.188 (0.105, 0.272) | 0.00021 |
| e99904945 | SpGSPGPDGKTGPpGP | P02452 | COL1A1 | Collagen alpha-1(I) chain | -0.186 (-0.270, -0.102) | 0.00027 |
| e99906617 | MpGSpGGpGSDGKpGpPG | P02461 | COL3A1 | Collagen alpha-1(III) chain | -0.172 (-0.255, -0.089) | 0.00029 |
| e99917288 | GPpGESGREGAPGAEGSpGRDGSpGAKGDRGETGp | P02452 | COL1A1 | Collagen alpha-1(I) chain | -0.184 (-0.267, -0.100) | 0.00031 |
| e99919885 | ERGEQGPAGSpGFQGLpGpAGppGEAGKpGEQGVPGDLGAPGPSG | P02452 | COL1A1 | Collagen alpha-1(I) chain | -0.179 (-0.261, -0.098) | 0.00034 |
| e99918864 | GpAGPPGPPGPPGTSGHPGSPGSPGYQGPPGEPGQAGPSGPPG | P02461 | COL3A1 | Collagen alpha-1(III) chain | -0.164 (-0.246, -0.082) | 0.00052 |
| e99904847 | GGDkGEDGDPGQPGp | P12107 | COL11A1 | Collagen alpha-1(XI) chain | 0.157 (0.074, 0.241) | 0.00063 |
| e99917866 | NTGApGSpGVSGPKGDAGQPGEKGSpGAQGPPGAPGPLG | P02461 | COL3A1 | Collagen alpha-1(III) chain | -0.166 (-0.250, -0.081) | 0.00070 |
| e99912508 | KGNSGEpGAPGSKGDTGAKGEpGpVG | P02452 | COL1A1 | Collagen alpha-1(I) chain | -0.176 (-0.260, -0.092) | 0.00073 |
| e99920509 | GEHGpPGPPGPIGPVGQPGAAGADGEPGARGPQGHFGAKGDEGTRGFNGP | P13942 | COL11A2 | Collagen alpha-2(XI) chain | -0.153 (-0.236, -0.070) | 0.00078 |
| e99915442 | DVStPPTVLPDNFPRYP | P01344 | IGF2 | Insulin-like growth factor II | 0.148 (0.066, 0.231) | 0.00088 |
| e99919425 | pGApGVRGFQGQKGSmGDpGLPGPQGLRGDVGDRGPGGAAGP | Q14050 | COL9A3 | Collagen alpha-3(IX) chain | 0.150 (0.066, 0.235) | 0.00089 |
| e99917787 | GARGLpGpPGSNGNPGPPGPSGSPGKDGPPGPAGNTGAP | P02461 | COL3A1 | Collagen alpha-1(III) chain | -0.160 (-0.243, -0.076) | 0.0011 |
| e99917280 | AAGEpGKAGERGVpGPpGAVGPAGKDGEAGAQGPPGP | P02452 | COL1A1 | Collagen alpha-1(I) chain | -0.169 (-0.253, -0.086) | 0.0012 |
| e99913779 | DDILASPPRLPEPQPYPGAPHHSS | P39060 | COL18A1 | Collagen alpha-1(XVIII) chain | 0.140 (0.059, 0.222) | 0.0012 |

**Supplementary Table 2.** Association of 99 bone-related sequenced urinary peptides with chronological age in the derivation dataset (continued)

| Peptide ID number | Amino-acid sequence | Parental proteins identified from the amino-acid sequence | | | β (95% confidence interval) | *p*-value |
|---|---|---|---|---|---|---|
| | | Accession number | Symbol | Name | | |
| e99917212 | RTGEVGAVGPpGFAGEKGPSGEAGTAGpPGTpGPQG | P08123 | COL1A2 | Collagen alpha-2(I) chain | -0.149 (-0.231, -0.067) | 0.0012 |
| e99909408 | GDDGEAGKpGRPGERGPPGp | P02452 | COL1A1 | Collagen alpha-1(I) chain | -0.166 (-0.247, -0.084) | 0.0013 |
| e99906961 | GLpGTGGPpGENGKPGEp | P02461 | COL3A1 | Collagen alpha-1(III) chain | -0.161 (-0.246, -0.075) | 0.0013 |
| e99914645 | DVStPPTVLPDNFPRYPVG | P01344 | IGF2 | Insulin-like growth factor II | 0.144 (0.061, 0.226) | 0.0014 |
| e99905423 | VGpPGPPGpPGPPGPPS | P02452 | COL1A1 | Collagen alpha-1(I) chain | -0.167 (-0.250, -0.084) | 0.0014 |
| e99905074 | DGHpGKpGRPGERG | P08123 | COL1A2 | Collagen alpha-2(I) chain | -0.149 (-0.232, -0.066) | 0.0014 |
| e99916419 | EAGRDGNpGNDGppGRDGQPGHKGERGYpG | P08123 | COL1A2 | Collagen alpha-2(I) chain | -0.146 (-0.227, -0.064) | 0.0016 |
| e99904531 | PGTpGSPGPAGASGNPG | P02458 | COL2A1 | Collagen alpha-1(II) chain | 0.147 (0.065, 0.228) | 0.0018 |
| e99916785 | DPGLMGERGEDGpAGNGTEGFpGFpGYPGNR | P12109 | COL6A1 | Collagen alpha-1(VI) chain | -0.137 (-0.221, -0.054) | 0.0020 |
| e99907513 | TGSpGSPGPDGKTGPpGPAG | P02452 | COL1A1 | Collagen alpha-1(I) chain | -0.164 (-0.247, -0.080) | 0.0022 |
| e99914815 | pGYPGQpGQDGKPGYQGIAGTpGVpGSPG | Q96P44 | COL21A1 | Collagen alpha-1(XXI) chain | -0.130 (-0.211, -0.049) | 0.0023 |
| e99917447 | GPpGDpGLMGERGEDGpAGNGTEGFPGFPGYPGN | P12109 | COL6A1 | Collagen alpha-1(VI) chain | -0.134 (-0.219, -0.049) | 0.0030 |
| e99911753 | GADGQPGAKGEPGDAGAKGDAGppGP | P02452 | COL1A1 | Collagen alpha-1(I) chain | -0.162 (-0.247, -0.077) | 0.0031 |
| e99918044 | AGGPGFPGApGAKGEAGpTGARGpEGAQGPRGEPGTPGS | P02458 | COL2A1 | Collagen alpha-1(II) chain | -0.143 (-0.227, -0.058) | 0.0039 |
| e99905705 | DGQPGAKGEpGDAGAKG | P02452 | COL1A1 | Collagen alpha-1(I) chain | -0.153 (-0.235, -0.071) | 0.0042 |
| e99906176 | VGPpGPpGPPGPpGPPSA | P02452 | COL1A1 | Collagen alpha-1(I) chain | -0.157 (-0.241, -0.073) | 0.0042 |
| e99915411 | PQGPpGPTGpGGDKGDTGPpGPQGLQGLpGT | P02461 | COL3A1 | Collagen alpha-1(III) chain | 0.144 (0.060, 0.228) | 0.0043 |
| e99918867 | QGFQGPAGEPGEPGQTGPAGARGPAGppGKAGEDGHpGKP | P08123 | COL1A2 | Collagen alpha-2(I) chain | -0.136 (-0.218, -0.053) | 0.0043 |
| e99914015 | PAGERGEQGAPGPSGFQGLPGPpGPPGE | P02458 | COL2A1 | Collagen alpha-1(II) chain | -0.140 (-0.224, -0.056) | 0.0048 |
| e99911879 | EPGPAGSKGESGNKGEPGSAGpQGp | P08123 | COL1A2 | Collagen alpha-2(I) chain | -0.138 (-0.222, -0.053) | 0.0049 |
| e99917184 | GPAGFAGPPGADGQPGAKGEQGEAGQKGDAGAPGPQG | P02458 | COL2A1 | Collagen alpha-1(II) chain | -0.136 (-0.219, -0.054) | 0.0050 |
| e99906686 | SpGNIGPAGKEGPVGLpG | P08123 | COL1A2 | Collagen alpha-2(I) chain | -0.132 (-0.215, -0.049) | 0.0065 |
| e99911537 | GKNGDDGEAGKPGRpGERGPpGP | P02452 | COL1A1 | Collagen alpha-1(I) chain | -0.153 (-0.238, -0.069) | 0.0067 |

**Supplementary Table 2.** Association of 99 bone-related sequenced urinary peptides with chronological age in the derivation dataset (continued)

| Peptide ID number | Amino-acid sequence | Parental proteins identified from the amino-acid sequence | | | β (95% confidence interval) | *p*-value |
|---|---|---|---|---|---|---|
| | | Accession number | Symbol | Name | | |
| e99910953 | DGQpGAKGEpGDAGAKGDAGPpGP | P02452 | COL1A1 | Collagen alpha-1(I) chain | 0.148 (0.066, 0.229) | 0.0068 |
| e99901648 | GpEGGKGAAGPpG | P02461 | COL3A1 | Collagen alpha-1(III) chain | 0.140 (0.054, 0.225) | 0.0078 |
| e99918881 | HDGEKGPRGKPGDMGPPGPQGPPGKDGPpGVKGE NGHp | Q5TAT6 | COL13A1 | Collagen alpha-1(XIII) chain | -0.118 (-0.203, -0.033) | 0.0085 |
| e99912556 | KNGDDGEAGKpGRPGERGpPGPQG | P02452 | COL1A1 | Collagen alpha-1(I) chain | -0.147 (-0.230, -0.064) | 0.0094 |
| e99902032 | GGpGSDGKpGPpG | P02461 | COL3A1 | Collagen alpha-1(III) chain | -0.134 (-0.217, -0.050) | 0.0095 |
| e99915467 | GRDGNpGNDGPpGRDGQpGHKGERGYpG | P08123 | COL1A2 | Collagen alpha-2(I) chain | -0.126 (-0.210, -0.043) | 0.0098 |
| e99919780 | GAPGERGETGPpGpAGFAGpPGADGQPGAKGEQGE AGQKGDAGAPG | P02458 | COL2A1 | Collagen alpha-1(II) chain | -0.128 (-0.212, -0.045) | 0.011 |
| e99915129 | ERGEAGIpGVpGAKGEDGKDGSPGEpGANG | P02461 | COL3A1 | Collagen alpha-1(III) chain | -0.129 (-0.210, -0.047) | 0.011 |
| e99906024 | SpGSPGPDGKTGpPGPAG | P02452 | COL1A1 | Collagen alpha-1(I) chain | -0.144 (-0.228, -0.061) | 0.012 |
| e99913707 | GApGQNGEPGGKGERGApGEKGEGGppG | P02461 | COL3A1 | Collagen alpha-1(III) chain | -0.129 (-0.212, -0.046) | 0.013 |
| e99908442 | AGEKGPSGEAGTAGPpGTpGP | P08123 | COL1A2 | Collagen alpha-2(I) chain | 0.124 (0.039, 0.208) | 0.014 |
| e99916417 | RTGEVGAVGPpGFAGEKGPSGEAGTAGPpGTpGP | P08123 | COL1A2 | Collagen alpha-2(I) chain | -0.123 (-0.208, -0.038) | 0.015 |
| e99911008 | pPGEEGKRGPRGDpGTVGPpGP | P05997 | COL5A2 | Collagen alpha-2(V) chain | -0.105 (-0.187, -0.022) | 0.016 |
| e99914315 | NRGERGSEGSPGHpGQpGPPGpPGApGP | P02461 | COL3A1 | Collagen alpha-1(III) chain | -0.127 (-0.210, -0.043) | 0.016 |
| e99913816 | ERGEAGIpGVpGAKGEDGKDGSpGEpG | P02461 | COL3A1 | Collagen alpha-1(III) chain | -0.128 (-0.212, -0.044) | 0.016 |
| e99901785 | DGESGRPGRpG | P02461 | COL3A1 | Collagen alpha-1(III) chain | 0.126 (0.043, 0.210) | 0.017 |
| e99905346 | GPpGKpGDDGEAGKpG | P02458 | COL2A1 | Collagen alpha-1(II) chain | 0.123 (0.039, 0.207) | 0.017 |
| e99908598 | NSGEPGApGSKGDTGAKGEp | P02452 | COL1A1 | Collagen alpha-1(I) chain | -0.138 (-0.220, -0.055) | 0.018 |
| e99902286 | PpGEAGKpGEQG | P02452 | COL1A1 | Collagen alpha-1(I) chain | -0.139 (-0.224, -0.055) | 0.020 |
| e99903284 | DGSpGAKGDRGET | P02452 | COL1A1 | Collagen alpha-1(I) chain | 0.138 (0.055, 0.222) | 0.021 |
| e99915811 | GHPGPSGPPGKpGYGSpGLQGEpGLPGPPGPS | Q03692 | COL10A1 | Collagen alpha-1(X) chain | -0.099 (-0.183, -0.016) | 0.021 |
| e99912156 | GPAGpPGEKGEPGDDGPSGAEGPPGp | P02458 | COL2A1 | Collagen alpha-1(II) chain | -0.117 (-0.200, -0.034) | 0.025 |
| e99902189 | GpPGPpGPpGPpG | P53420 | COL4A4 | Collagen alpha-4(IV) chain | -0.096 (-0.180, -0.012) | 0.025 |

**Supplementary Table 2.** Association of 99 bone-related sequenced urinary peptides with chronological age in the derivation dataset (ends)

| Peptide ID number | Amino-acid sequence | Parental proteins identified from the amino-acid sequence | | | β (95% confidence interval) | *p*-value |
|---|---|---|---|---|---|---|
| | | Accession number | Symbol | Name | | |
| e99906650 | EGSpGRDGSpGAKGDRG | P02452 | COL1A1 | Collagen alpha-1(I) chain | -0.129 (-0.209, -0.049) | 0.028 |
| e99913065 | ADGQpGAKGEpGDAGAKGDAGPpGPAGP | P02452 | COL1A1 | Collagen alpha-1(I) chain | -0.128 (-0.208, -0.048) | 0.029 |
| e99917672 | GPpGpAGFAGpPGADGQPGAKGEQGEAGQKGDAG APGP | P02458 | COL2A1 | Collagen alpha-1(II) chain | -0.116 (-0.201, -0.032) | 0.029 |
| e99904330 | pPGpPGPpGpPGPPS | P02452 | COL1A1 | Collagen alpha-1(I) chain | -0.132 (-0.216, -0.048) | 0.034 |
| e99909989 | DGESGRpGRpGERGLpGPpG | P02461 | COL3A1 | Collagen alpha-1(III) chain | 0.118 (0.034, 0.203) | 0.034 |
| e99905551 | VGPpGPPGPpGPpGPPS | P02452 | COL1A1 | Collagen alpha-1(I) chain | -0.133 (-0.218, -0.049) | 0.035 |
| e99905559 | GpAGpRGERGPPGESG | P08123 | COL1A2 | Collagen alpha-2(I) chain | -0.110 (-0.196, -0.025) | 0.0371 |
| e99920326 | GSEGARGAPGPAGPPGDPGLMGERGEDGPAGNGT EGFpGFpGYpGNR | P12109 | COL6A1 | Collagen alpha-1(VI) chain | -0.094 (-0.179, -0.010) | 0.043 |
| e99918041 | NTGAPGSpGVSGpKGDAGQpGEKGSpGAQGppGAP GPLG | P02461 | COL3A1 | Collagen alpha-1(III) chain | -0.115 (-0.200, -0.030) | 0.044 |
| e99906697 | VGPpGpPGPpGPPGPPSAG | P02452 | COL1A1 | Collagen alpha-1(I) chain | -0.129 (-0.213, -0.044) | 0.046 |

FLEMENGHO: Flemish Study on Environment, Genes, and Health Outcomes. The lower-case letters in the amino-acid sequence identify post-translational modifications: c, disulphide bridges; m, oxidized methionine; n, deaminated asparagine; p, hydroxylated proline; k, hydroxylated lysine; q, deaminated glutamine. Accession numbers refer to the Uniprot database (http://www.uniprot.org/uniprot). Association sizes (β) between urinary peptides and chronological age were expressed per 10-year increment and were adjusted for the clustering within families, sex, body mass index, the glomerular filtration rate calculated from serum creatinine by the Chronic Kidney Disease Epidemiology Collaboration equation, current smoking, γ-glutamyltransferase, fasting plasma glucose, physical activity, use of diuretics (yes *vs* no), and socioeconomic status (High or middle *vs* low). *p*-values were adjusted for multiple testing using the Benjamini-Hochberg false discovery rate for 395 tested associations.

**Supplementary Table 3.** Association of chronological age with 16 bone-related urinary peptides

| Peptide ID number | Parental protein | Derivation dataset (*n* = 519) | | Time-shifted validation dataset (*n* = 519) | | Synchronous validation dataset (*n* = 187) | |
|---|---|---|---|---|---|---|---|
| | | β (95% CI) | *p*-value | β (95% CI) | *p*-value | β (95% CI) | *p*-value |
| e99907132 | COL1A1 | 0.281 (0.203, 0.359) | 9.21E-11 | 0.293 (0.205, 0.381) | 1.51E-09 | 0.151 (0.028, 0.274) | 0.093 |
| e99910072 | COL3A1 | -0.232 (-0.309, -0.155) | 3.29E-08 | -0.171 (-0.260, -0.082) | 0.00059 | -0.201 (-0.329, -0.073) | 0.026 |
| e99901132 | MGP | 0.230 (0.154, 0.305) | 3.85E-08 | 0.232 (0.146, 0.318) | 8.67E-07 | 0.238 (0.113, 0.363) | 0.0007 |
| e99904196 | COL2A1 | 0.237 (0.156, 0.318) | 7.19E-08 | 0.134 (0.042, 0.226) | 0.0098 | NS | … |
| e99919107 | COL1A2 | -0.218 (-0.297, -0.139) | 3.00E-07 | -0.106 (-0.198, -0.015) | 0.034 | -0.218 (-0.343, -0.093) | 0.0028 |
| e99904847 | COL11A1 | 0.157 (0.074, 0.241) | 0.00063 | NS | … | NS | … |
| e99920509 | COL11A2 | -0.153 (-0.236, -0.070) | 0.00078 | NS | … | NS | … |
| e99915442 | IGF2 | 0.148 (0.066, 0.231) | 0.00088 | 0.100 (0.005, 0.194) | 0.04 | 0.160 (0.026, 0.294) | 0.028 |
| e99919425 | COL9A3 | 0.150 (0.066, 0.235) | 0.00089 | 0.129 (0.037, 0.221) | 0.011 | NS | … |
| e99913779 | COL18A1 | 0.140 (0.059, 0.222) | 0.0012 | 0.113 (0.023, 0.203) | 0.018 | NS | … |
| e99916785 | COL6A1 | -0.137 (-0.221, -0.054) | 0.0020 | NS | | -0.140 (-0.276, -0.004) | 0.05 |
| e99914815 | COL21A1 | -0.130 (-0.211, -0.049) | 0.0023 | -0.100 (-0.189, -0.010) | 0.033 | NS | … |
| e99918881 | COL13A1 | -0.118 (-0.203, -0.033) | 0.0085 | NS | … | -0.134 (-0.268, -0.001) | 0.049 |
| e99911008 | COL5A2 | -0.105 (-0.187, -0.022) | 0.016 | NS | … | -0.178 (-0.316, -0.040) | 0.019 |
| e99915811 | COL10A1 | -0.099 (-0.183, -0.016) | 0.021 | NS | … | -0.179 (-0.312, -0.047) | 0.016 |
| e99902189 | COL4A4 | -0.096 (-0.180, -0.012) | 0.025 | NS | … | -0.145 (-0.278, -0.012) | 0.041 |

Only urinary peptides with the highest significance per identified protein were selected for this analysis to demonstrate consistency of the associations in 519 participants included in the derivation and the time-shifted validation datasets and in the 187 participants included in the synchronous validation dataset. Association sizes (β) of chronological age with the urinary peptides were expressed per 10-year increment and were adjusted for the clustering within families, sex, body mass index, the glomerular filtration rate calculated from serum creatinine by the Chronic Kidney Disease Epidemiology Collaboration equation, current smoking, γ-glutamyltransferase, fasting plasma glucose, physical activity, use of diuretics (yes *vs* no), and socioeconomic status (high or middle *vs* low). The *p*-values indicate the validation status ($p < 0.10$, given the prior probability established by the significance in the derivation dataset). NS indicates not significant.

**Supplementary Table 4.** Number of replicated bone-related urinary peptides identifying parental proteins

| **Peptide ID number** | **Parental protein** | **Number of peptide fragments (Percentage replicated compared with derivation dataset)** | | |
|---|---|---|---|---|
| | | **Derivation dataset (*n* = 519)** | **Time-shifted validation dataset (*n* = 519)** | **Synchronous validation dataset (*n* = 187)** |
| e99907132 | COL1A1 | 38 | 16 (42.1%) | 19 (50%) |
| e99910072 | COL3A1 | 21 | 10 (47.6%) | 6 (28.6%) |
| e99901132 | MGP | 2 | 1 (50%) | 2 (100%) |
| e99904196 | COL2A1 | 10 | 4 (40%) | 5 (50%) |
| e99919107 | COL1A2 | 14 | 3 (21.4%) | 8 (57.1%) |
| e99904847 | COL11A1 | 1 | 0 (0%) | 0 (0%) |
| e99920509 | COL11A2 | 1 | 0 (0%) | 0 (0%) |
| e99915442 | IGF2 | 2 | 2 (100%) | 1 (50%) |
| e99919425 | COL9A3 | 1 | 1 (100%) | 0 (0%) |
| e99913779 | COL18A1 | 1 | 1 (100%) | 0 (0%) |
| e99916785 | COL6A1 | 3 | 0 (0%) | 1 (33.3%) |
| e99914815 | COL21A1 | 1 | 1 (100%) | 1 (100%) |
| e99918881 | COL13A1 | 1 | 0 (0%) | 0 (0%) |
| e99911008 | COL5A2 | 1 | 0 (0%) | 1 (100%) |
| e99915811 | COL10A1 | 1 | 0 (0%) | 1 (100%) |
| e99902189 | COL4A4 | 1 | 0 (0%) | 1 (100%) |

Replication indicates similar directionality in the association with chronological age with a *p-value* of <0.10 (given the prior probability established by the significance in the derivation dataset).

**Supplementary Table 5.** Sex difference of bone-related urinary peptides in the derivation dataset (starts)

| Peptide ID number | Parental protein | Unadjusted | | Adjusted | |
|---|---|---|---|---|---|
| | | Estimate (95% CI) | *p-value* | Estimate (95% CI) | *p-value* |
| e99918573 | COL1A2 | -0.551 (-0.716, -0.386) | 1.39E-08 | -0.490 (-0.663, -0.317) | 4.42E-06 |
| e99915129 | COL3A1 | -0.544 (-0.709, -0.379) | 1.25E-08 | -0.496 (-0.671, -0.320) | 2.49E-06 |
| e99919107 | COL1A2 | -0.483 (-0.650, -0.316) | 7.38E-07 | -0.464 (-0.634, -0.295) | 3.10E-06 |
| e99915778 | COL1A1 | -0.440 (-0.608, -0.273) | 7.26E-06 | -0.324 (-0.497, -0.151) | 0.0021 |
| e99909408 | COL1A1 | -0.426 (-0.594, -0.258) | 1.47E-05 | -0.357 (-0.533, -0.181) | 0.00077 |
| e99917184 | COL2A1 | -0.424 (-0.592, -0.256) | 1.42E-05 | -0.375 (-0.553, -0.198) | 0.00043 |
| e99913707 | COL3A1 | -0.419 (-0.587, -0.251) | 1.63E-05 | -0.403 (-0.582, -0.225) | 0.00016 |
| e99915811 | COL10A1 | -0.418 (-0.586, -0.250) | 1.59E-05 | -0.435 (-0.614, -0.256) | 5.03E-05 |
| e99908598 | COL1A1 | -0.394 (-0.562, -0.226) | 5.59E-05 | -0.379 (-0.557, -0.202) | 0.00041 |
| e99915411 | COL3A1 | -0.373 (-0.542, -0.204) | 0.00016 | -0.321 (-0.501, -0.140) | 0.0037 |
| e99903016 | COL1A1 | -0.355 (-0.524, -0.186) | 0.00037 | -0.281 (-0.458, -0.105) | 0.0091 |
| e99905074 | COL1A2 | -0.329 (-0.499, -0.160) | 0.001 | -0.295 (-0.473, -0.117) | 0.0068 |
| e99910072 | COL3A1 | -0.316 (-0.485, -0.146) | 0.0017 | -0.256 (-0.436, -0.077) | 0.019 |
| e99914315 | COL3A1 | -0.314 (-0.484, -0.145) | 0.0017 | -0.243 (-0.409, -0.077) | 0.016 |
| e99911008 | COL5A2 | -0.311 (-0.480, -0.141) | 0.0019 | -0.238 (-0.416, -0.060) | 0.03 |
| e99906686 | COL1A2 | -0.307 (-0.477, -0.138) | 0.002 | -0.313 (-0.492, -0.133) | 0.004 |
| e99913779 | COL18A1 | -0.291 (-0.461, -0.121) | 0.0036 | -0.266 (-0.441, -0.091) | 0.013 |
| e99912508 | COL1A1 | -0.287 (-0.457, -0.117) | 0.0041 | -0.278 (-0.458, -0.097) | 0.012 |
| e99916419 | COL1A2 | -0.284 (-0.454, -0.114) | 0.0045 | -0.274 (-0.450, -0.099) | 0.01 |
| e99906650 | COL1A1 | -0.271 (-0.441, -0.101) | 0.0072 | NS | … |

**Supplementary Table 5.** Sex difference of bone-related urinary peptides in the derivation dataset (ends)

| Peptide ID number | Parental protein | Unadjusted | | Adjusted | |
|---|---|---|---|---|---|
| | | Estimate (95% CI) | *p-value* | Estimate (95% CI) | *p-value* |
| e99904945 | COL1A1 | -0.254 (-0.424, -0.084) | 0.013 | NS | … |
| e99911777 | COL1A1 | -0.245 (-0.415, -0.074) | 0.016 | -0.239 (-0.418, -0.060) | 0.031 |
| e99913816 | COL3A1 | -0.240 (-0.411, -0.069) | 0.017 | NS | … |
| e99911753 | COL1A1 | -0.240 (-0.411, -0.070) | 0.018 | -0.242 (-0.424, -0.060) | 0.03 |
| e99917866 | COL3A1 | -0.232 (-0.402, -0.061) | 0.023 | NS | … |
| e99918041 | COL3A1 | 0.207 (0.036, 0.378) | 0.048 | NS | … |
| e99915442 | IGF2 | 0.209 (0.038, 0.380) | 0.046 | 0.261 (0.085, 0.438) | 0.016 |
| e99906709 | COL1A1 | 0.243 (0.072, 0.413) | 0.017 | 0.215 (0.047, 0.382) | 0.039 |
| e99901661 | COL2A1 | 0.248 (0.078, 0.419) | 0.015 | 0.306 (0.133, 0.478) | 0.0034 |
| e99903120 | COL1A1 | 0.250 (0.079, 0.420) | 0.014 | 0.282 (0.102, 0.463) | 0.011 |
| e99900340 | MGP | 0.260 (0.090, 0.430) | 0.011 | 0.308 (0.135, 0.481) | 0.0036 |
| e99918044 | COL2A1 | 0.305 (0.135, 0.474) | 0.0021 | 0.260 (0.078, 0.441) | 0.019 |
| e99913065 | COL1A1 | 0.311 (0.141, 0.480) | 0.0018 | 0.332 (0.160, 0.505) | 0.0015 |
| e99906441 | COL1A1 | 0.323 (0.154, 0.493) | 0.0013 | 0.313 (0.143, 0.482) | 0.0024 |
| e99901132 | MGP | 0.340 (0.170, 0.509) | 0.00068 | 0.372 (0.210, 0.535) | 0.00015 |
| e99910953 | COL1A1 | 0.342 (0.173, 0.512) | 0.00063 | 0.285 (0.109, 0.460) | 0.0083 |
| e99905560 | COL1A1 | 0.465 (0.298, 0.632) | 1.81E-06 | 0.475 (0.306, 0.643) | 1.7E-06 |

NS: not significant. Estimate was the association size of difference in urinary peptide level in women compared to men (men as reference) derived from the rank-normalized data in the unadjusted and adjusted general linear models. Adjusted models accounted for the clustering within families, age, body mass index, the glomerular filtration rate calculated from serum creatinine by the Chronic Kidney Disease Epidemiology Collaboration equation, current smoking, γ-glutamyltransferase as index of alcohol intake, blood glucose, physical activity, use of diuretics (yes *vs.* no), and the middle or high socioeconomic status (yes vs. no). The 99 urinary peptides associated with chronological age were included in the analysis in the derivation dataset. *P-values* were corrected for the Benjamini-Hochberg false discovery rate for 99 tested association. 37 (9 parental proteins) was identified in the unadjusted models and 32 urinary peptides (9 parental proteins) in the adjusted models, respectively.

**Supplementary Table 6.** Weights assigned by elastic net regression to the multidimensional UPP biomarker reflecting aging (starts)

| Peptide ID number | Amino-acid sequence | Parental proteins identified from the amino-acid sequence | | | Weight (95% confidence interval) |
|---|---|---|---|---|---|
| | | Accession number | Symbol | Name | |
| … | … | … | … | … | 49.577405 (intercept) |
| e99911879 | EPGPAGSKGESGNKGEPGSAGpQGp | P08123 | COL1A2 | Collagen alpha-2(I) chain | -0.006570 (-0.010338, -0.002746) |
| e99919107 | DQGPVGRTGEVGAVGPpGFAGEKGpSGEAGTAGPPG TpGPQG | P08123 | COL1A2 | Collagen alpha-2(I) chain | -0.005577 (-0.008878, -0.002536) |
| e99905705 | DGQPGAKGEpGDAGAKG | P02452 | COL1A1 | Collagen alpha-1(I) chain | -0.005512 (-0.013123, 0.001801) |
| e99902032 | GGpGSDGKpGPpG | P02461 | COL3A1 | Collagen alpha-1(III) chain | -0.005088 (-0.010774, 0.000741) |
| e99902189 | GpPGPpGPpGPpG | P53420 | COL4A4 | Collagen alpha-4(IV) chain | -0.005073 (-0.008518, -0.001569) |
| e99916966 | PpGESGREGApGAEGSpGRDGSPGAKGDRGETGP | P02452 | COL1A1 | Collagen alpha-1(I) chain | -0.004863 (-0.006168, -0.003606) |
| e99903372 | DGQPGAKGEpGDAG | P02452 | COL1A1 | Collagen alpha-1(I) chain | -0.004531 (-0.010864, 0.001061) |
| e99910072 | GLpGTGGPpGENGKpGEpGPKG | P02461 | COL3A1 | Collagen alpha-1(III) chain | -0.004197 (-0.007435, -0.001032) |
| e99906650 | EGSpGRDGSpGAKGDRG | P02452 | COL1A1 | Collagen alpha-1(I) chain | -0.003875 (-0.009837, 0.002081) |
| e99906709 | GSpGSpGPDGKTGPpGPAG | P02452 | COL1A1 | Collagen alpha-1(I) chain | -0.003650 (-0.004616, -0.002698) |
| e99912851 | LDGAKGDAGPAGPKGEpGSpGENGApG | P02452 | COL1A1 | Collagen alpha-1(I) chain | -0.003648 (-0.006470, -0.000989) |
| e99912949 | LDGAKGDAGPAGpKGEpGSpGENGApG | P02452 | COL1A1 | Collagen alpha-1(I) chain | -0.003329 (-0.008020, 0.001176) |
| e99920509 | GEHGpPGPPGPIGPVGQPGAAGADGEPGARGPQGHF GAKGDEGTRGFNGP | P13942 | COL11A2 | Collagen alpha-2(XI) chain | -0.001102 (-0.001807, -0.000415) |
| e99906441 | TGSpGSpGPDGKTGPpGP | P02452 | COL1A1 | Collagen alpha-1(I) chain | -0.000776 (-0.002173, 0.000571) |
| e99905560 | GSpGSpGPDGKTGPPGp | P02452 | COL1A1 | Collagen alpha-1(I) chain | -0.000259 (-0.000961, 0.000439) |
| e99914645 | DVStPPTVLPDNFPRYPVG | P01344 | IGF2 | Insulin-like growth factor II | 0.000179 (0.000003, 0.000347) |
| e99915442 | DVStPPTVLPDNFPRYP | P01344 | IGF2 | Insulin-like growth factor II | 0.000350 (-0.000203, 0.000945) |

**Supplementary Table 6.** Weights assigned by elastic net regression to the multidimensional UPP biomarker reflecting aging (ends).

| Peptide ID number | Amino-acid sequence | Parental proteins identified from the amino-acid sequence | | | Weight (95% confidence interval) |
|---|---|---|---|---|---|
| | | Accession number | Symbol | Name | |
| e99901132 | cDDYRLcE | P08493 | MGP | Matrix Gla protein | 0.000822 (0.000486, 0.001196) |
| e99909989 | DGESGRpGRpGERGLpGPpG | P02461 | COL3A1 | Collagen alpha-1(III) chain | 0.001280 (0.000333, 0.002376) |
| e99907132 | EpGSpGENGAPGQmGPR | P02452 | COL1A1 | Collagen alpha-1(I) chain | 0.001361 (0.000879, 0.001887) |
| e99913779 | DDILASPPRLPEPQPYPGAPHHSS | P39060 | COL18A1 | Collagen alpha-1(XVIII) chain | 0.002118 (-0.002625, 0.007012) |
| e99904196 | PSGDQGASGpAGPSGP | P02458 | COL2A1 | Collagen alpha-1(II) chain | 0.002342 (-0.001725, 0.006138) |
| e99910953 | DGQpGAKGEpGDAGAKGDAGPpGP | P02452 | COL1A1 | Collagen alpha-1(I) chain | 0.002584 (0.001634, 0.003548) |
| e99903284 | DGSpGAKGDRGET | P02452 | COL1A1 | Collagen alpha-1(I) chain | 0.002860 (-0.000424, 0.005988) |
| e99908442 | AGEKGPSGEAGTAGPpGTpGP | P08123 | COL1A2 | Collagen alpha-2(I) chain | 0.002947 (0.000610, 0.005217) |
| e99905346 | GPpGKpGDDGEAGKpG | P02458 | COL2A1 | Collagen alpha-1(II) chain | 0.004666 (0.002066, 0.007324) |
| e99917280 | AAGEpGKAGERGVpGPpGAVGPAGKDGEAGAQGPPG P | P02452 | COL1A1 | Collagen alpha-1(I) chain | 0.005564 (0.003320, 0.007863) |
| e99916417 | RTGEVGAVGPpGFAGEKGPSGEAGTAGPpGTpGP | P08123 | COL1A2 | Collagen alpha-2(I) chain | 0.005706 (0.003379, 0.008284) |
| e99919425 | pGApGVRGFQGQKGSmGDpGLPGPQGLRGDVGDRGP GGAAGP | Q14050 | COL9A3 | Collagen alpha-3(IX) chain | 0.010065 (0.004812, 0.014979) |

The derivation dataset included the baseline data of 519 participants in FLEMENGHO. Accession numbers refer to the Uniprot database (http://www.uniprot.org/uniprot). Association sizes (β) between chronological age and the levels of the urinary peptides were derived by elastic net regression, which was bootstrapped 1000 times. The regression slopes (βs) reflect the weight of each of the 29 peptide fragments in the construction of the multidimensional UPPost-age biomarker. Of 99 peptides entering the data reduction analysis, 29 entered in at least 60% of models and identified 10 proteins. The 95% confidence intervals of the βs were derived from the bootstrap distribution.

**Supplementary Table 7.** Risk of having HASD ≤ -3 cm in FLEMENGHO participants at baseline (2005-2010).

| Variable | n/N | Chronological age | | UPPost-age | |
|---|---|---|---|---|---|
| | | OR (95% CI) | *p*-value | OR (95% CI) | *p*-value |
| All participants | 278/706 | 1.43 (1.25, 1.63) | <0.0001 | 1.31 (1.12, 1.53) | 0.0007 |
| Men | 188/349 | 1.40 (1.17, 1.67) | 0.0002 | 1.19 (0.96, 1.48) | 0.11 |
| Women | 90/357 | 1.51 (1.21, 1.88) | 0.0003 | 1.43 (1.14, 1.81) | 0.0024 |
| Postmenopausal women | 62/183 | 1.57 (1.06, 2.34) | 0.025 | 1.22 (0.88, 1.70) | 0.24 |

Abbreviations: HASD: the difference of body height minus arm span; n/N: number of participants with HASD ≤ -3 cm/total number of participants; UPPost-age: age as predicted by the urinary proteomic profile; OR: odds ratio; 95% CI: 95% confidence interval. ORs, given with 95% CI, express the relative risk per 10-year increment in chronological age or UPPost-age with adjustment for clustering within families, sex (in all participants only), current smoking, γ-glutamyltransferase, fasting plasma glucose, physical activity, use of diuretics (yes *vs* no) and socioeconomic status (high and middle *vs* low).

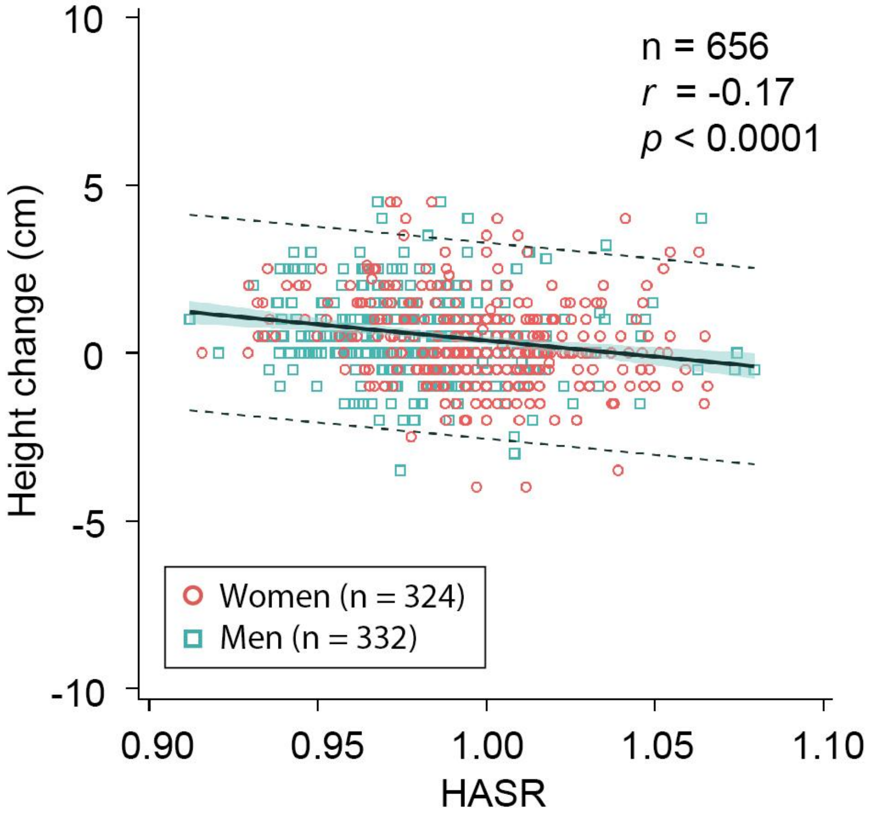


**Supplementary Figure 1. Correlation between height loss and body-height-to-arm-span ratio in 656 FLEMENGHO participants.** HASR: body-height-to-arm-span ratio. *r* is the Pearson correlation coefficient. Height change was the differences between two body height measurement examined in the interval from 1985 until 2008. During the median time interval of 9.56 years (interquartile range: 6.54, 15.5 years), the mean height change was 0.46 cm (SD: 1.51 cm) in 656 participants (50.6% women; median age at the first measurement: 40.5 year). Height change was significantly correlated with HASR ($r$ = -0.17, $p < 0.0001$). The sex-specific correlation coefficients were -0.24 in women (red circles) and -0.095 in men (green squares), respectively ($p$ = 0.057 for the sex difference).

# SUPPLEMENTARY DATA

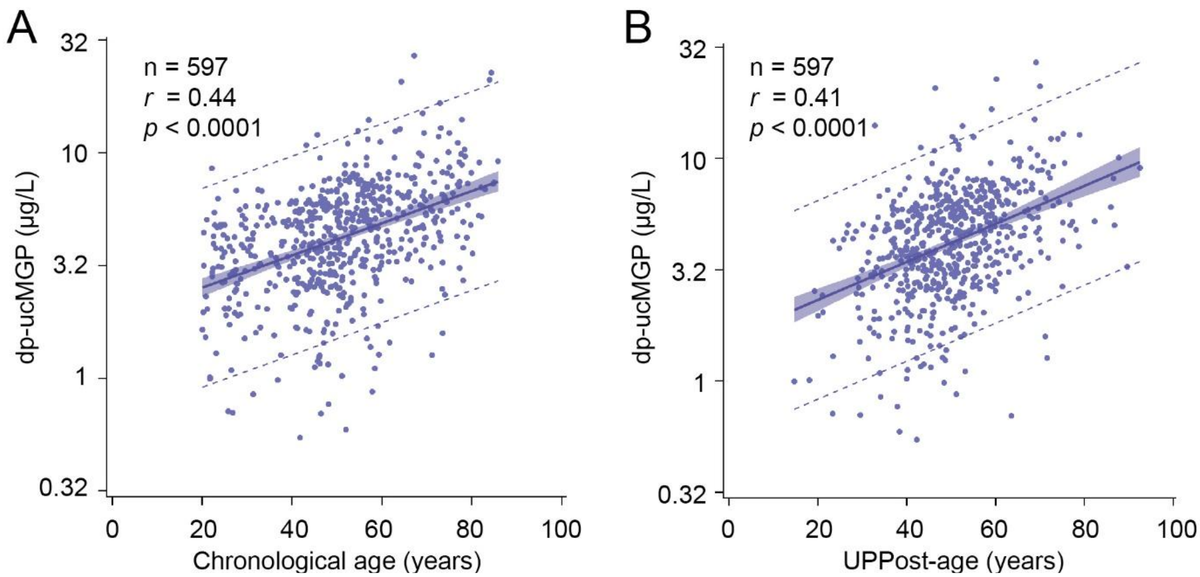


**Supplementary Figure 2. Regression plot of dp-ucMGP with chronological age (A) and UPPost-age (B) in 597 FLEMENGHO participants.** *r* is the Pearson correlation coefficient. dp-ucMGP denotes desphospho-uncarboxylated matrix Gla protein. UPPost-age is age as predicted by the urinary proteomic profile.

© 2024. Yu YL et al. Published online at http://www.aginganddisease.org/EN/10.14336/AD.2024.0303

# SUPPLEMENTARY DATA

© 2024. Yu YL et al. Published online at http://www.aginganddisease.org/EN/10.14336/AD.2024.0303
